# MRI-Based Radiomics Model for Pretreatment Prediction of Pathological Lymph Node Metastasis in Neuroendocrine Carcinoma of the Cervix: Dual-Center Development and External Validation

**DOI:** 10.3390/curroncol33100625

**Published:** 2026-10-16

**Authors:** Jian Chen, Fengle Jiang, Xingfa Chen, Rong Lin, Ning Ma, Haijuan Yu, Yang Sun

**Affiliations:** 1 Department of Gynecology, Clinical Oncology School of Fujian Medical University, Fujian Cancer Hospital, Fuzhou 350014, China; marsz3@126.com (J.C.); yuhaijuan_fjmu@163.com (H.Y.); 2 Innovation Center for Cancer Research, Clinical Oncology School of Fujian Medical University, Fujian Cancer Hospital, Fuzhou 350014, China; 13763800713@163.com; 3 Fujian Key Laboratory of Advanced Technology for Cancer Screening and Early Diagnosis, Fuzhou 350014, China; 4 Department of Radiology, Clinical Oncology School of Fujian Medical University, Fujian Cancer Hospital, Fuzhou 350014, China; xingfanihao@163.com (X.C.); 15059452552@163.com (N.M.); 5 Department of Obstetrics and Gynecology, Fujian Provincial Hospital, Fuzhou 350001, China; 1310306093lr@fjmu.edu.cn

**Keywords:** neuroendocrine carcinoma of the cervix, lymph node metastasis, magnetic resonance imaging, radiomics, external validation

## Abstract

**Objectives:** To develop and externally validate MRI-based radiomics and clinicoradiomics models for predicting pathological lymph node metastasis (LNM) in neuroendocrine carcinoma of the cervix (NECC). **Methods:** This retrospective two-center study included 133 patients who underwent pretreatment pelvic MRI and pathological nodal assessment: 93 for development and 40 for external validation. Intratumoral and peritumoral features were extracted from T2-weighted, diffusion-weighted, and contrast-enhanced T1-weighted images. Stratified nested fivefold cross-validation evaluated the complete revised selection process. Locked models were assessed for discrimination, calibration, and exploratory net benefit, with conventional MRI nodal assessment as a comparator. **Results:** Pathological LNM was present in 54/133 patients (40.6%). The selected radiomics-only k-nearest neighbors (KNN) model had an external AUC of 0.783 (95% CI, 0.630 to 0.935), accuracy of 0.800, sensitivity of 0.813, and specificity of 0.792. The complete selection procedure yielded an out-of-fold AUC of 0.731. External AUCs were 0.727 for the clinical/MRI model and 0.767 for the clinicoradiomics model; neither fusion model differed significantly from the clinical/MRI comparator. Conventional MRI sensitivity was 0.563, with no significant paired difference from the selected model (*p* = 0.125). External calibration was imperfect (Brier score, 0.186; slope, 0.672). **Conclusions:** MRI radiomics showed moderate external discrimination of pathological LNM in NECC. Adding clinical variables did not demonstrate further benefit. Multicenter validation, recalibration, and clinical-impact assessment are needed before clinical application.

## Simple Summary

Neuroendocrine carcinoma of the cervix is a rare, aggressive cancer. Knowing whether it has spread to lymph nodes helps guide treatment, but routine scans can miss this spread. We studied 133 patients from two hospitals to assess whether computer analysis of magnetic resonance imaging (MRI) scans could estimate the risk of lymph node spread before pathological nodal assessment. Using data from one hospital, we developed models that combined measurements from the tumor and surrounding tissue, with and without clinical information. We then tested the models using data from the other hospital. The selected model combined tumor and surrounding-tissue measurements. Adding clinical information did not clearly improve prediction, and the external comparisons did not show that the model performed better than the clinical/MRI model or conventional MRI nodal assessment. The approach may help assess lymph node spread in this rare cancer, but larger studies are needed before clinical use.

## 1. Introduction

Neuroendocrine carcinoma of the cervix (NECC) is a rare, highly aggressive cervical cancer comprising mainly small-cell and large-cell neuroendocrine carcinomas [1,2]. Like small-cell lung cancer, it shows rapid proliferation and early hematogenous and lymphatic spread [3]. Patients often present with regional lymph node involvement, distant metastasis, or both. Most retrospective series report substantially worse outcomes than those for squamous cell carcinoma or adenocarcinoma at comparable stages [4]. Evidence comes largely from retrospective cohorts and expert consensus, and management generally combines systemic chemotherapy with definitive local therapy [2,5,6].

Lymph node metastasis (LNM) has prognostic and treatment implications and is associated with increased relapse risk in NECC [7,8]. The 2018 International Federation of Gynecology and Obstetrics (FIGO) system classifies pelvic and para-aortic nodal metastases as stages IIIC1 and IIIC2, respectively [9]. The suffixes “r” and “p” indicate imaging- and pathology-based assignments. Nodal status can influence the extent of nodal assessment and radiotherapy planning [10].

Pelvic magnetic resonance imaging (MRI) is widely used to stage cervical cancer because of its high soft-tissue contrast and multiparametric capability [11,12]. Conventional nodal assessment relies on short-axis diameter, shape, margin, and necrosis. These morphologic features have limited sensitivity for micrometastases and do not reliably distinguish reactive enlargement from metastasis [12,13]. Meta-analyses report an MRI sensitivity of approximately 0.58 for nodal metastasis despite relatively high specificity [14]. Fluorodeoxyglucose positron emission tomography/computed tomography (FDG PET/CT) may improve diagnostic performance, but access is limited in some settings and small-volume disease remains difficult to detect [13,14,15,16]. Sentinel lymph node mapping has reduced surgical morbidity in selected patients with common cervical cancer histologies [10,17,18]. Its role in NECC remains uncertain because disease-specific evidence is scarce.

MRI radiomics quantifies features beyond visual assessment and has been studied for preoperative LNM prediction in cervical cancer [19]. Systematic reviews and meta-analyses have examined the diagnostic performance of MRI-based radiomics and other imaging artificial intelligence approaches for this purpose [20,21,22]. Studies have also combined radiomic features with clinical variables to improve pretreatment risk assessment [21,23]. Most, however, concern squamous cell carcinoma and adenocarcinoma. Their findings cannot be assumed to generalize to NECC, which differs in metastatic behavior and treatment approach [1,2]. If clinical benefit is established, these models could help identify patients needing further nodal evaluation when conventional MRI findings are equivocal.

Radiogenomic studies have reported associations between imaging phenotypes and molecular or immune features [24,25], and multiomic profiling has demonstrated substantial biological heterogeneity in small-cell neuroendocrine carcinoma of the cervix [26]. We used matched tumor samples from a small subset of our cohort to explore candidate imaging-transcriptomic associations, without establishing molecular mechanisms.

We aimed to develop and externally validate pretreatment MRI-based radiomics and clinicoradiomics models for predicting pathological LNM in untreated patients with pathologically confirmed NECC. We constructed intratumoral and peritumoral signatures from routine multiparametric pelvic MRI alongside pretreatment clinical and MRI-derived variables that excluded nodal status. FIGO 2018 stage was excluded to avoid label leakage. We also explored patient-level associations between regional Rad-scores and tumor microenvironmental programs using single-nucleus RNA sequencing of matched formalin-fixed paraffin-embedded tumor samples.

## 2. Methods

### 2.1. Study Design and Patients

**Figure 1:**
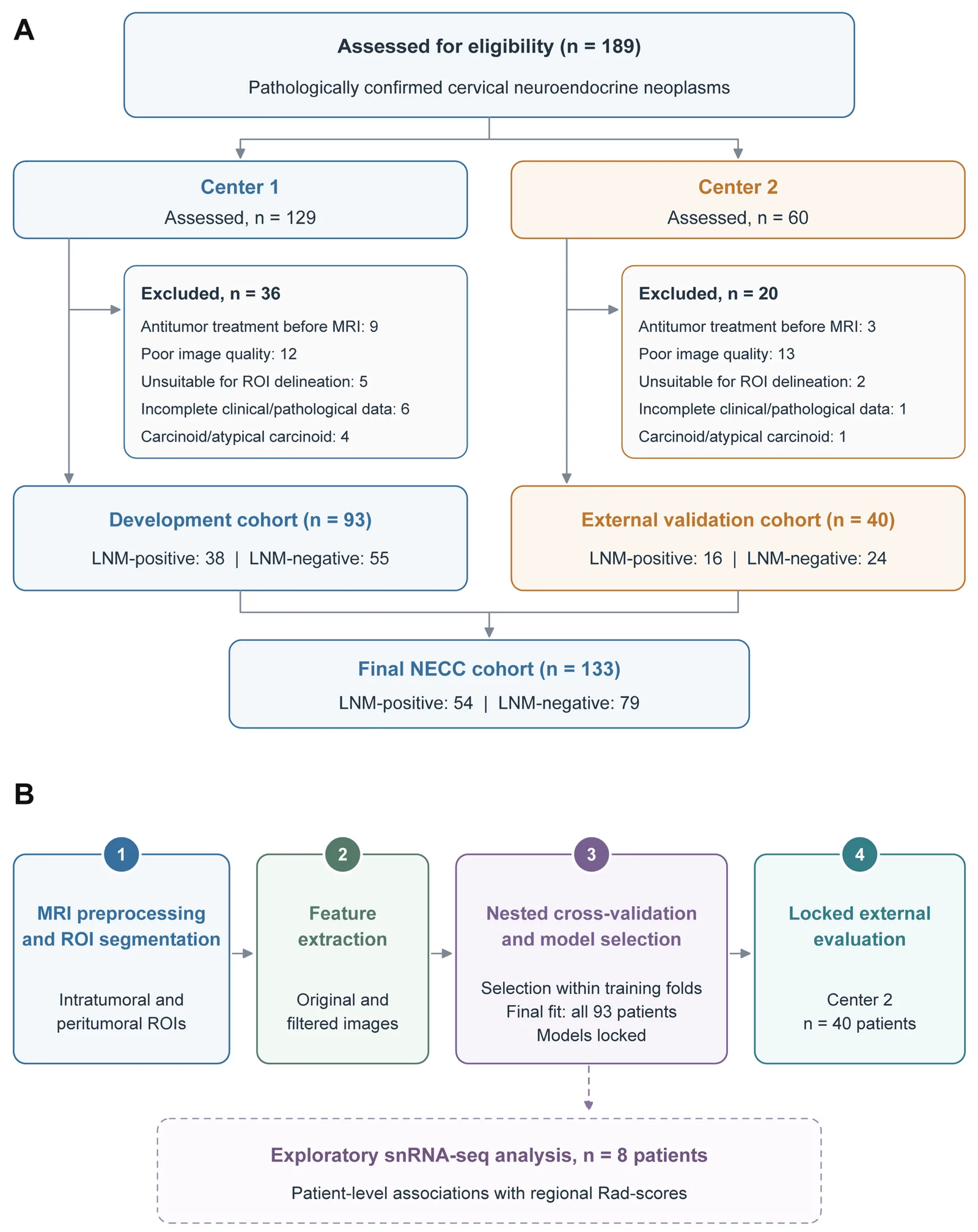
Participant flow, study design, and radiomics analytic workflow. (**A**) Center-specific screening, exclusions, and final cohorts. Of 189 patients with pathologically confirmed cervical neuroendocrine neoplasms assessed for eligibility, 133 patients with NECC were included: 93 for development and 40 for external validation. (**B**) Simplified radiomics workflow. Feature selection and model tuning were performed within training folds during nested cross-validation. Final models were fitted to all 93 development patients and locked before external evaluation. Exploratory snRNA-seq analysis examined associations with regional Rad-scores in eight matched patients. LNM, lymph node metastasis; MRI, magnetic resonance imaging; NECC, neuroendocrine carcinoma of the cervix; ROI, region of interest; snRNA-seq, single-nucleus RNA sequencing.

This retrospective study assessed consecutive patients with pathologically confirmed cervical neuroendocrine neoplasms who underwent lymphadenectomy or nodal sampling, with or without definitive primary tumor resection. Patients were assessed at Fujian Medical University Cancer Hospital (Center 1) and Fujian Provincial Hospital (Center 2) between 14 July 2007 and 4 May 2024. Patients meeting the NECC eligibility criteria formed the final cohort: 93 from Center 1 for development and 40 from Center 2 for external validation. Figure 1A shows participant flow at each center, and Figure 1B shows the study design and radiomics workflow.

Eligibility criteria were: (i) age 18–80 years; (ii) histologically confirmed NECC, including small-cell or large-cell neuroendocrine carcinoma; (iii) pretreatment abdominopelvic MRI within 2 weeks before nodal tissue acquisition; and (iv) available pathological nodal status, required MRI sequences for radiomics extraction, and prespecified pretreatment clinical/MRI predictors. Exclusion criteria were: (i) neoadjuvant chemoradiotherapy or other antitumor treatment before MRI; (ii) motion artifacts, DWI distortion, or insufficient spatial resolution that prevented reliable tumor delineation or radiomics analysis; (iii) tumors too small or with margins too indistinct for reliable region of interest (ROI) delineation; (iv) incomplete key clinical or pathological data; and (v) well-differentiated neuroendocrine tumors, including cervical carcinoid or atypical carcinoid. Both MRI and the nodal tissue acquisition preceded antitumor treatment; patients undergoing surgery after neoadjuvant chemotherapy were excluded. Radiologists blinded to pathological nodal status assessed image quality.

The Ethics Committee of Fujian Cancer Hospital approved the study (K2021-043-01) and waived informed consent for the retrospective analysis of clinical and MRI data. All eight patients in the tissue-based analysis provided written biobank consent for research use of residual samples and associated clinical information, including tumor-related genetic research. External data were de-identified and provided under institutional collaboration and data-sharing procedures.

### 2.2. Endpoint, Nodal Assessment, and Candidate Predictors

The primary endpoint was pathological LNM, defined as metastatic involvement of at least one resected or sampled lymph node. All patients who underwent radical surgery also underwent pelvic lymphadenectomy. Other patients underwent nodal sampling or selective excision without primary tumor resection; some enlarged nodes were removed with therapeutic intent. Assessment involved pelvic and/or para-aortic nodes. A negative nodal label indicated that no metastasis was detected in the examined nodes. Supplementary Table S15A reports positive and negative counts by center for systematic lymphadenectomy and selective nodal excision or biopsy only. The Supplementary Methods (File S1) describe assessment and treatment pathways. Conventional MRI nodal assessment served as a clinical comparator and was excluded from the candidate predictors.

FIGO 2009 staging [27] was used because FIGO 2018 incorporates nodal status [9], risking label leakage. Stage was assigned from pretreatment gynecologic examination, biopsy histology, and MRI only. For patients treated from 2018 onward, it was reconstructed without nodal status or postoperative pathology. Coding was 0 for IA–IIA2 and 1 for IIB–IVB.

Candidate pretreatment predictors included age, maximum tumor diameter, FIGO 2009 stage, biopsy-reported mixed histology, MRI-assessed signs of local invasion, and inflammatory and nutritional indices from pretreatment blood tests. Invasion signs comprised parametrial, vaginal, uterine corpus, vesicouterine space, and rectouterine space involvement. Definitions, coding, and excluded variables are detailed in the Supplementary Methods and Supplementary Table S1.

### 2.3. MRI Acquisition and Interpretation

Abdominopelvic MRI was performed using 1.5-T or 3.0-T scanners with phased-array body coils: Signa 1.5T EXCITE III HD (GE Healthcare, Milwaukee, WI, USA), Discovery 750w 3.0T (GE Healthcare, Waukesha, WI, USA), and Prisma 3.0T (Siemens Healthcare GmbH, Erlangen, Germany). Axial T2-weighted imaging (T2WI), axial diffusion-weighted imaging (DWI; b = 800 s/mm^2^), and contrast-enhanced T1-weighted imaging (T1WI-CE) were exported in DICOM format. All patients at both centers received gadoterate meglumine (Beijing Beilu Pharmaceutical Co., Ltd., Beijing, China; 0.2 mL/kg), and acquisition of the T1WI-CE images used for radiomics began approximately 2 min after injection started. Supplementary Table S2 provides acquisition parameters for each scanner and sequence and the scanner distribution by center. Apparent diffusion coefficient (ADC) maps informed clinical nodal assessment but were excluded from all radiomics models under the prespecified three-sequence workflow.

Two radiologists with 11 and 20 years of experience in pelvic MRI independently reviewed the pretreatment images while blinded to pathological outcomes and resolved disagreements by consensus. Before model development, they recorded MRI-assessed signs of local invasion for evaluation as candidate clinical/MRI predictors. Their final binary assessment of nodal metastasis, based on the criteria in the Supplementary Methods, was compared with the radiomics-only and clinicoradiomics models.

### 2.4. Tumor Segmentation and Radiomics Workflow

DWI and T1WI-CE were rigidly registered to axial T2WI, with visual quality checks. All sequences were resampled to isotropic 1.0 mm voxels using linear interpolation, followed by N4 bias-field correction and per-volume z-score intensity normalization [28]. Segmentation masks were resampled using nearest-neighbor interpolation. During revision, features were re-extracted from these existing normalized images with PyRadiomics internal normalization disabled and the fixed bin width changed from 25 to 0.05. The revised width was chosen on technical grounds, considering gray-level occupancy, extraction completeness, and computational feasibility; external-validation performance was not used for selection. Feature and classifier selection and radiomics and fusion model fitting were repeated (Section 2.5). Supplementary Table S3 provides preprocessing and feature-extraction settings.

Primary tumor volumes of interest were manually delineated slice by slice on axial T2WI using 3D Slicer version 5.8.1 [29], with DWI and T1WI-CE as additional references for tumor boundaries. Readers were blinded to pathological LNM status. The tumor volume included cystic, necrotic, and hemorrhagic components. Peritumoral annuli were created by morphologically dilating the tumor volume by 3 mm and 5 mm and subtracting the original volume. Annuli were anatomically edited on T2WI using predefined rules and propagated to co-registered DWI and T1WI-CE.

Features were extracted using PyRadiomics version 3.1.0 and a fixed YAML configuration file [30]. Feature definitions and terminology followed the Image Biomarker Standardisation Initiative where applicable [31]. Extraction included shape, first-order, and texture features from original and filtered images. Interobserver reproducibility was assessed using paired delineations from 20 development-cohort patients, with intratumoral and peritumoral features assessed separately. The revised pipeline retained features with a finite intraclass correlation coefficient (ICC) ≥0.85, using the two-way random-effects, absolute-agreement, single-measurement ICC(A,1). During cross-validation, ICCs were calculated using only repeat-segmented patients in the corresponding training partition.

### 2.5. Model Development and External Validation

The preprocessing steps above were applied identically to both cohorts. All data-adaptive procedures were restricted to the corresponding training partitions: ICC screening, ROI and sequence selection, feature filtering and selection, classifier tuning, and model fitting. Stratified nested cross-validation used five outer and five inner folds. Pooled outer-fold predictions provided out-of-fold (OOF) estimates for each model-family procedure and for the complete procedure including family selection; these did not evaluate one fixed final model. Final models were fitted to all 93 development patients and locked before external evaluation, without external refitting, recalibration, threshold modification, or reselection.

After constant features were removed and features were standardized within training partitions, highly correlated features were removed by Spearman correlation filtering. Recursive feature elimination (RFE) and LASSO linear regression with fivefold cross-validation followed. LASSO selected the regularization parameter and nonzero coefficients for continuous Rad-scores, which were classifier inputs rather than probabilities. Features, scaling parameters, and formulas were applied unchanged to held-out patients; Supplementary Table S4 reports feature counts.

The three-predictor clinical/MRI logistic model from the initial analysis of the 93 development patients was retained as a fixed comparator. Existing predictions were used without refitting; no corresponding nested OOF estimate was available. Eight classifiers were evaluated for radiomics and fusion: logistic regression, support vector machine (SVM), k-nearest neighbors (KNN), decision tree, random forest, XGBoost, LightGBM, and Gaussian naive Bayes. Hyperparameter searches used grids in the Supplementary Methods and mean inner-fold AUC for selection. No synthetic oversampling was applied. For clinicoradiomics fusion, LASSO selected clinical variables from 13 pretreatment candidates within each training partition. Fusion inputs were standardized using that partition’s parameters.

Seven sequence combinations across intratumoral, 3 mm peritumoral, and 5 mm peritumoral regions yielded 21 configurations. Within each outer training partition, inner-fold AUC determined the best intratumoral and peritumoral configurations. Fusion evaluated only this regional pair, with and without selected clinical variables, using the same classifier families.

The reanalysis replaced the initial intratumoral T2WI and 5 mm peritumoral T2WI + DWI signatures with intratumoral T1WI-CE (Rad-score_Tumor) and 3 mm peritumoral T1WI-CE + DWI (Rad-score_Peritumor) signatures, respectively. Regional classifiers were SVM and KNN, respectively; both fusion models used KNN. Supplementary Table S5 gives the score formulas, scaling parameters, clinical coding, and classifier specifications. Accompanying model files support reproduction of the final predictions, and the Supplementary Methods detail the searches and fitting procedures.

### 2.6. Model Evaluation

Discrimination was assessed using the area under the receiver operating characteristic curve (AUC) with DeLong 95% confidence intervals for external estimates [32]. Apparent development and nested OOF performance are reported separately. Accuracy, sensitivity, specificity, positive and negative predictive values, balanced accuracy, and confusion matrices used a probability threshold of 0.50, applied unchanged to both cohorts. Classification metrics are accompanied by Wilson intervals where reported. All 10 pairwise external AUC comparisons were exploratory and used paired DeLong tests with Holm adjustment. Conventional MRI was compared with both fusion models using two-sided exact McNemar tests for sensitivity, specificity, and accuracy, without multiplicity adjustment.

Calibration used logistic spline curves, Brier scores, and jointly estimated logistic calibration intercepts and slopes. Confidence intervals used 1000 patient-level bootstrap resamples with predictive models held fixed. Decision curve analysis compared radiomics-only fusion with treat-all and treat-none strategies [33]. The model-directed strategy specified intensified pretreatment nodal evaluation and multidisciplinary review above a selected threshold. These thresholds were exploratory and had not been validated as clinical cutoffs.

Interpretability analyses used the KNN probability surface and exact marginal probability-scale SHapley Additive exPlanations (SHAP). All 93 development patients formed the background for external-prediction explanations (Supplementary Methods).

### 2.7. Exploratory Radiomics–Transcriptomics Analysis

The matched formalin-fixed paraffin-embedded (FFPE) single-nucleus RNA sequencing (snRNA-seq) subset comprised eight patients. Patient-level cell proportions and mean gene set variation analysis (GSVA) pathway scores [34] were correlated separately with the locked intratumoral T1WI-CE and 3 mm peritumoral T1WI-CE + DWI Rad-scores; fusion-model probabilities were not used. Analyses covered five major cell classes and 38 subtypes. Missing pathway values were not imputed; correlations required paired measurements in at least seven patients. The Supplementary Methods detail proportion denominators, barcode matching, and missing measurements.

Two-sided Spearman *p* values were calculated by exact enumeration of patient-label permutations, retaining observed tied ranks. Benjamini–Hochberg correction used all eligible cell-proportion and pathway correlations across both annotation levels within each regional Rad-score, including non-significant and undisplayed comparisons. The 11 previously selected cell–pathway pairs remained post hoc descriptive examples (Supplementary Table S13). Transcriptomic_Source_Data.zip (File S2) provides paired measurements, effective sample sizes, and complete results. These analyses were exploratory and did not establish links between the selected transcriptional programs and pathological LNM.

### 2.8. Statistical Analysis

Continuous variables are reported as mean ± standard deviation and categorical variables as number (%). Between-cohort comparisons used Welch’s *t* test for age, Mann–Whitney U tests for other continuous variables, Fisher’s exact test for histologic subtype, and Pearson χ^2^ tests for other categorical variables. Absolute standardized mean differences described balance between cohorts. All candidate predictors were complete in the final modeling cohort, so no imputation was performed. Tests were two-sided. Model development used Python version 3.6.15, and descriptive statistical analyses used R version 4.3.1. Software versions for the transcriptomic reanalysis, independent verification, and final figure generation are detailed in the Supplementary Methods. A retrospective descriptive subgroup analysis evaluated the locked radiomics-only model and conventional MRI among patients who underwent systematic lymphadenectomy, separately for each center. Existing predictions were used, with the model probability threshold fixed at 0.50 and no model refitting. We report patient counts, confusion matrices, and classification metrics with 95% Wilson confidence intervals. Development model results were identified as apparent performance (Supplementary Table S15B).

## 3. Results

### 3.1. Patient Characteristics

Of 189 consecutively assessed patients with pathologically confirmed cervical neuroendocrine neoplasms, 133 with NECC were included after 56 exclusions. The development and external validation cohorts comprised 93 and 40 patients, respectively (Figure 1A). Image-quality exclusions accounted for 12 of 129 patients assessed at Center 1 (9.3%) and 13 of 60 at Center 2 (21.7%). Pathological LNM was present in 54 of 133 patients (40.6%): 38 of 93 (40.9%) in the development cohort and 16 of 40 (40.0%) in the external validation cohort.

Small-cell neuroendocrine carcinoma accounted for 126 of 133 cases (94.7%), and biopsy-reported mixed histology was identified in 48 patients (36.1%). Table 1 summarizes baseline characteristics by cohort. At Center 1, 74 patients underwent systematic lymphadenectomy (30 LNM positive and 44 negative), and 19 underwent selective nodal excision or biopsy only (8 positive and 11 negative). At Center 2, 29 patients underwent systematic lymphadenectomy (8 positive and 21 negative), and 11 underwent selective nodal excision or biopsy only (8 positive and 3 negative). The 14 patients with negative findings after selective nodal excision or biopsy comprised seven with FIGO 2009 stage III disease and seven with stage IV disease (Supplementary Table S15A).

### 3.2. Clinical/MRI Model

The retained clinical/MRI model included MRI-assessed uterine corpus involvement, maximum tumor diameter, and FIGO 2009 stage. Its apparent development AUC was 0.749, and its external AUC was 0.727 (95% CI, 0.561 to 0.893; Table 2).

### 3.3. Intratumoral and Peritumoral Radiomics Signatures

**Figure 2:**
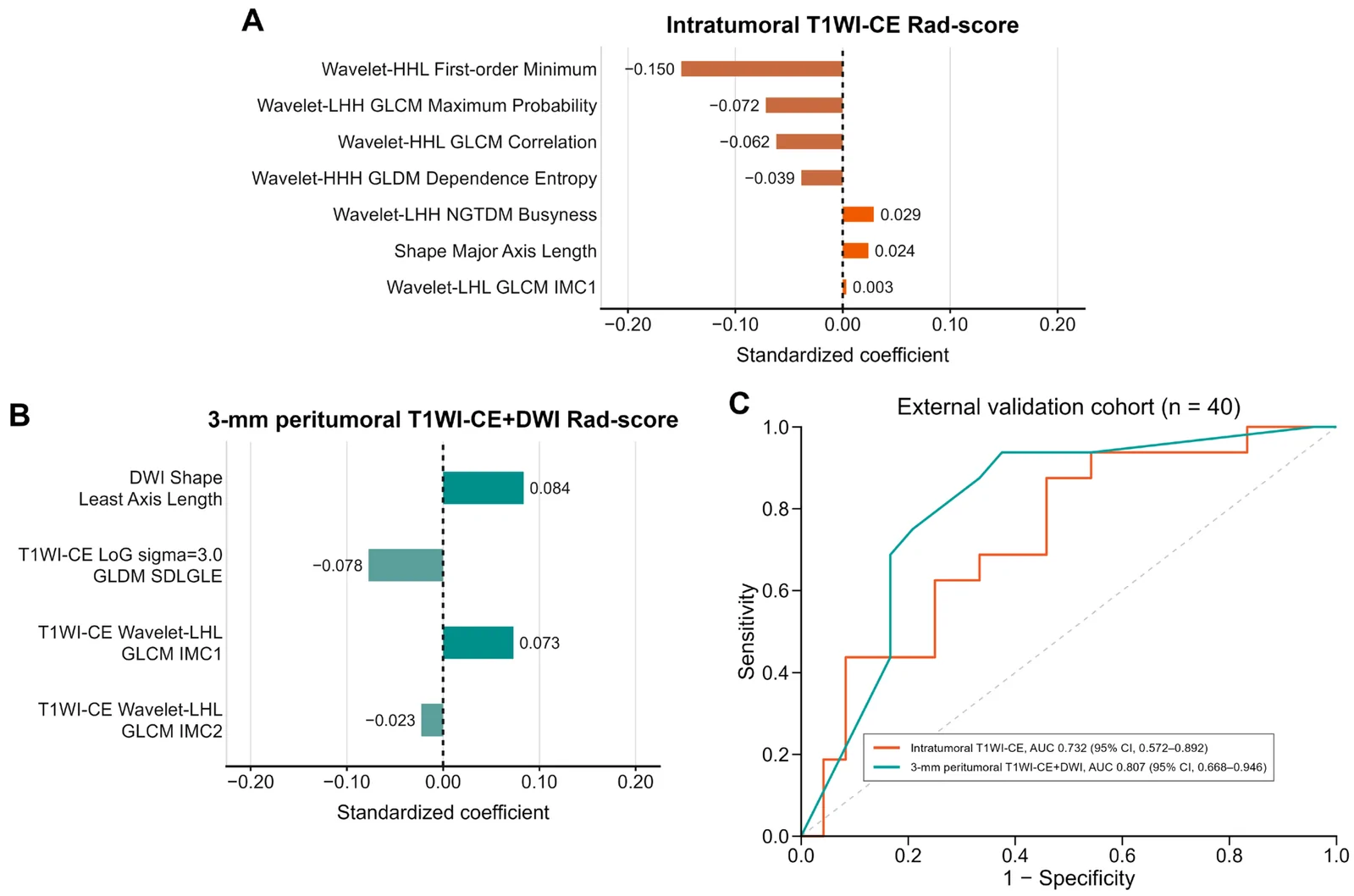
Regional radiomics signatures and external predictive performance. (**A**,**B**) LASSO coefficients for standardized features comprising the intratumoral T1WI-CE and 3 mm peritumoral T1WI-CE + DWI signatures, respectively. The black dashed vertical lines in panels A and B indicate zero coefficients. Complete Rad-score formulas are provided in Supplementary Table S5. (**C**) External validation ROC curves for the intratumoral SVM and peritumoral KNN classifiers (*n* = 40), with AUCs and DeLong 95% confidence intervals. The gray dashed diagonal in panel C indicates chance discrimination (AUC = 0.50). Orange and teal denote intratumoral and peritumoral signatures, respectively. T1WI-CE, contrast-enhanced T1-weighted imaging; DWI, diffusion-weighted imaging; LNM, lymph node metastasis; SVM, support vector machine; KNN, k-nearest neighbors; ROC, receiver operating characteristic; AUC, area under the ROC curve.

The intratumoral T1WI-CE signature retained seven features and used a sigmoid-kernel SVM classifier (Figure 2A). Its apparent development AUC was 0.859 and its external validation AUC was 0.732 (95% CI, 0.572 to 0.892; Table 2). The intratumoral development procedure yielded a pooled OOF AUC of 0.689.

The 3 mm peritumoral T1WI-CE + DWI signature selected during development retained four features and used KNN with 17 uniformly weighted neighbors (Figure 2B). Its apparent development AUC was 0.862, and its external AUC was 0.807 (95% CI, 0.668 to 0.946; Table 2). The peritumoral development procedure yielded an OOF AUC of 0.621. Although its external AUC was numerically highest (Table 2), external performance did not determine model selection. Selected feature coefficients and external ROC curves are shown in Figure 2A–C; Rad-score distributions and apparent development ROC curves are provided in Supplementary Figure S1A,B. Supplementary Table S7 summarizes classifier benchmarking.

### 3.4. Radiomics-Only and Clinicoradiomics Models

**Figure 3:**
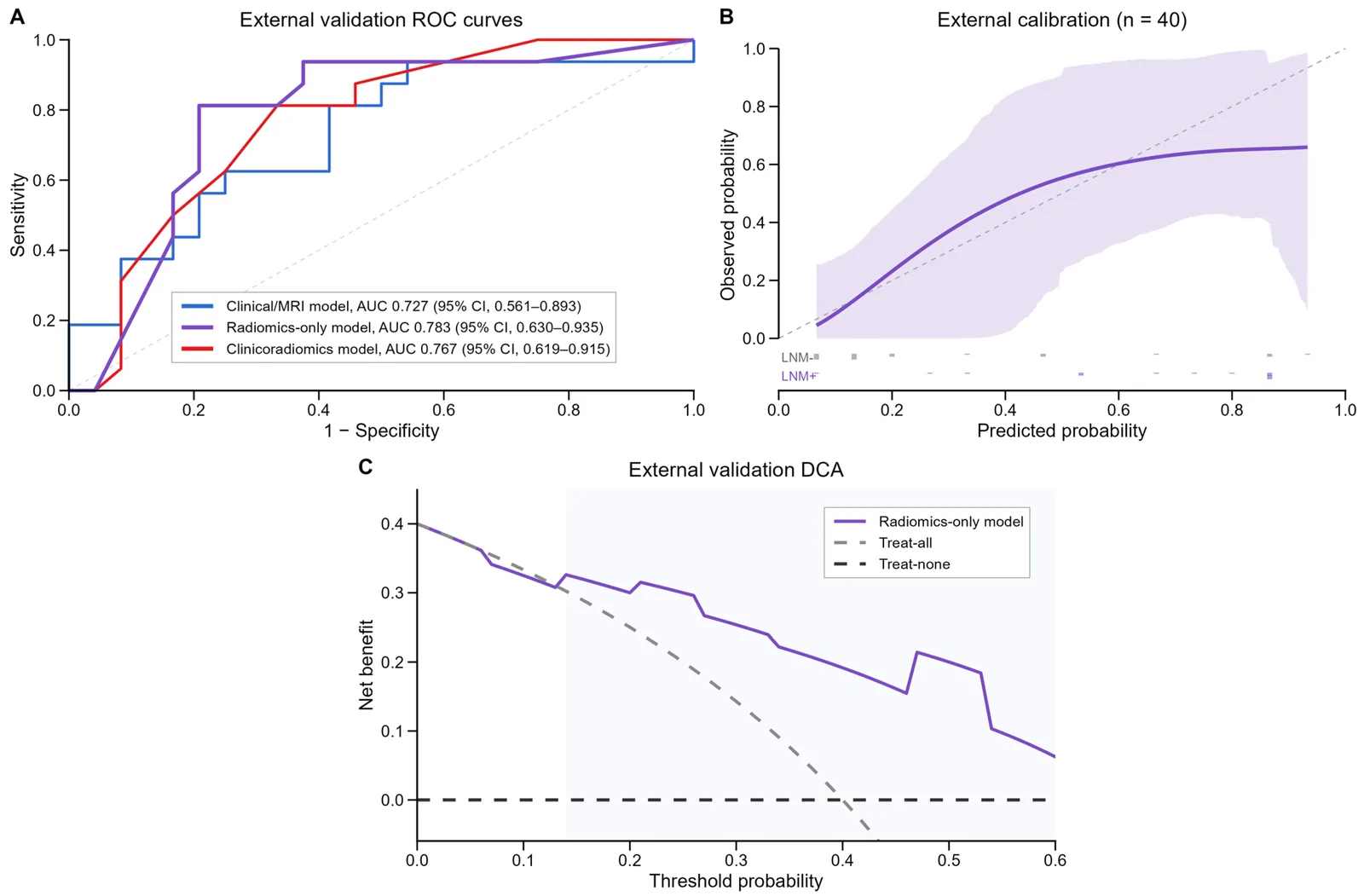
Model discrimination, calibration, and decision curve analysis (*n* = 40). (**A**) ROC curves for the clinical/MRI, radiomics-only fusion, and clinicoradiomics models, with AUCs and DeLong 95% confidence intervals. The gray dashed diagonal in panel A indicates chance discrimination (AUC = 0.50). (**B**) Calibration of the radiomics-only KNN fusion model. The curve uses logistic regression with a three-knot restricted cubic spline of logit-transformed predicted probability. The band indicates pointwise 95% confidence intervals from 1000 patient bootstrap samples, conditional on the fitted model. The dashed diagonal indicates ideal calibration; rug marks show individual predicted probabilities (LNM-negative, upper gray row; LNM-positive, lower purple row). The Brier score was 0.186 (bootstrap 95% CI, 0.110 to 0.279). The jointly estimated calibration intercept and slope were −0.346 (bootstrap 95% CI, −1.183 to 0.511) and 0.672 (bootstrap 95% CI, 0.228 to 1.478), respectively. (**C**) Decision curves for the radiomics-only model versus treat-all and treat-none. The gray dashed line represents treat-all, and the black horizontal dashed line represents treat-none (zero net benefit). Shading marks evaluated thresholds ≥0.05 where observed model net benefit exceeds both references. ROC, receiver operating characteristic; AUC, area under the ROC curve; KNN, k-nearest neighbors; LNM, lymph node metastasis.

The selected radiomics-only model combined Rad-score_Tumor and Rad-score_Peritumor using KNN with 15 uniformly weighted neighbors. Its apparent development AUC was 0.876 (Supplementary Figure S2A), and its external AUC was 0.783 (95% CI, 0.630 to 0.935; Figure 3A). External accuracy, sensitivity, and specificity were 0.800, 0.813, and 0.792, respectively (Table 2). Corresponding Wilson 95% confidence intervals were 0.652–0.895, 0.570–0.934, and 0.595–0.908. Its external AUC did not differ significantly from the clinical/MRI model (unadjusted *p* = 0.333).

OOF AUCs were 0.713 for the fusion-family procedure and 0.731 for the complete procedure including selection among model families. Radiomics-only fusion was selected in four of the five outer folds, but none selected the final configuration combining intratumoral T1WI-CE and 3 mm peritumoral T1WI-CE + DWI scores using KNN. Because regional configurations and classifiers varied across folds, these estimates evaluate the development procedures, not one fixed final KNN model (Supplementary Table S16).

The clinicoradiomics model combined both Rad-scores with seven clinical variables using KNN with 15 uniformly weighted neighbors. These variables were parametrial, uterine corpus, and vesicouterine space involvement, maximum tumor diameter, log10-transformed systemic immune-inflammation index (SII), prognostic nutritional index (PNI), and binary FIGO 2009 stage. Its apparent development AUC was 0.867, and its external AUC was 0.767 (95% CI, 0.619 to 0.915); the clinicoradiomics development procedure yielded an OOF AUC of 0.708. Its external AUC did not differ significantly from the clinical/MRI model (unadjusted *p* = 0.492) or radiomics-only fusion (*p* = 0.711). All 10 pairwise external AUC comparisons remained non-significant after Holm adjustment. Supplementary Table S8 provides confusion matrices at the fixed threshold of 0.50.

In external validation, conventional MRI sensitivity, specificity, and accuracy were 0.563, 0.833, and 0.725, respectively (Supplementary Table S14). The selected radiomics-only model had numerically higher sensitivity and accuracy but lower specificity than conventional MRI. The paired *p* values were 0.125, 1.000, and 0.453 for sensitivity, specificity, and accuracy, respectively. Differences between clinicoradiomics and MRI were also non-significant. Complete comparisons, including apparent development performance, are provided in Supplementary Table S14.

The systematic lymphadenectomy subgroup comprised 74 patients from the development cohort and 29 from the external validation cohort. In the external subgroup, the radiomics-only model had sensitivity and specificity of 75.0% (6/8) and 76.2% (16/21), compared with 37.5% (3/8) and 81.0% (17/21) for conventional MRI. The locked radiomics-only model had an AUC of 0.717 in the external systematic lymphadenectomy subgroup. Supplementary Table S15B provides full subgroup metrics, 95% confidence intervals, and confusion matrices; development results represent apparent performance. These estimates are descriptive because the external subgroup included only eight LNM-positive patients and differed in case mix, including stage distribution, from the full external cohort.

### 3.5. Calibration, Clinical Utility, and Model Interpretability

In the development cohort, the selected radiomics-only model had an apparent Brier score of 0.132; its jointly estimated calibration intercept and slope were 0.132 and 1.085, respectively (Supplementary Figure S2B). In external validation, the Brier score was 0.186 (95% bootstrap CI, 0.110 to 0.279), the intercept was −0.346 (−1.183 to 0.511), and the slope was 0.672 (0.228 to 1.478). Calibration was imperfect and estimates remained uncertain (Figure 3B). The clinicoradiomics model had an external Brier score of 0.203. Its slope of 0.037 was sensitive to an LNM-negative patient assigned a probability of 1.000 and to the subsequent logit clipping.

In external decision curve analysis, the selected radiomics-only model had higher net benefit than treat-all and treat-none at thresholds of 0.20, 0.30, and 0.40 (Figure 3C). Apparent development decision curves are provided in Supplementary Figure S2C. The peritumoral-only model had numerically higher net benefit at these thresholds. These descriptive comparisons do not validate clinical cutoffs or establish improved patient outcomes.

**Figure 4:**
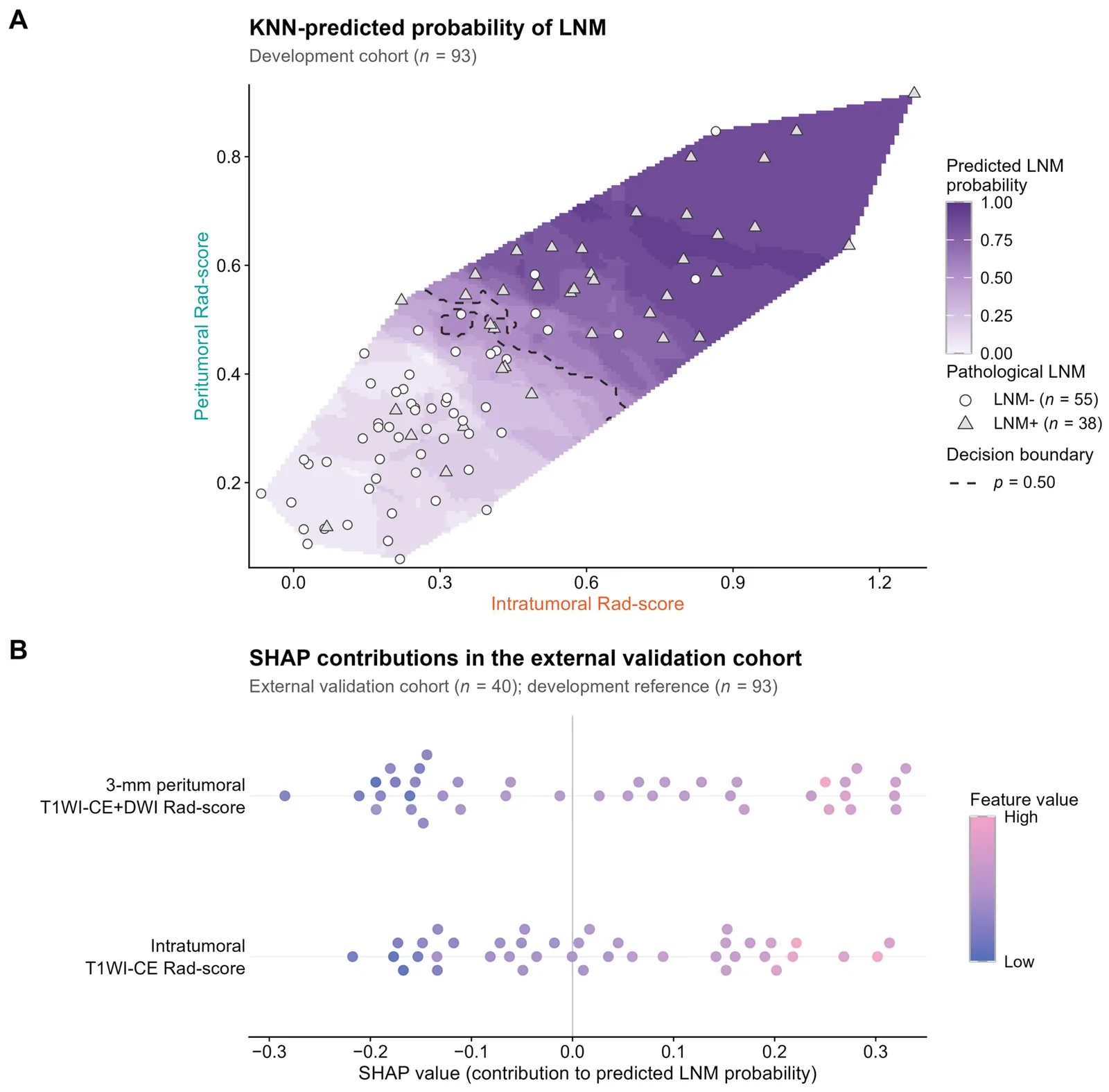
Prediction surface and SHAP explanations of the radiomics-only KNN fusion model. (**A**) Predicted LNM probability across the two regional Rad-scores, restricted to the development-data convex hull without smoothing. Circles and triangles indicate LNM-negative (*n* = 55) and LNM-positive (*n* = 38) patients, respectively. The dashed line approximates the classification boundary at a probability threshold of 0.50. (**B**) Probability-scale SHAP contributions for 40 external patients, using all 93 development patients as the background reference. Each point represents one patient. Positive contributions increase predicted probability relative to the reference expectation of 0.395. Rows are ordered by mean absolute SHAP value; colors indicate feature values, scaled separately for each Rad-score. SHAP describes model predictions, not causal effects. SHAP, SHapley Additive exPlanations.

Figure 4A shows the KNN probability surface. For external patients, the mean absolute probability-scale SHAP contribution was larger for Rad-score_Peritumor (0.175) than for Rad-score_Tumor (0.127). Higher regional Rad-scores generally shifted predictions toward higher LNM probability (Figure 4B). These contributions describe the fitted model under the development-cohort reference distribution; they do not measure independent or causal effects.

### 3.6. Exploratory Radiomics–Transcriptomics Associations

The matched subset included five patients with pathological LNM and three without LNM. Cell-type and subtype annotation and composition are shown in Supplementary Figures S3, S4 and S5A,B. None of the 86 cell-proportion correlations reached unadjusted exact *p* < 0.05, and no eligible cell-proportion or pathway association survived Benjamini–Hochberg correction at q < 0.05. The 11 previously displayed cell-pathway pairs are retained as post hoc descriptive examples in Supplementary Figure S5C,D and Supplementary Table S13. Individual paired measurements and complete correlation results are provided in Transcriptomic_Source_Data.zip (File S2) to permit verification. These findings do not establish biological mechanisms or transcriptional differences between LNM groups.

## 4. Discussion

In this two-center study, the radiomics-only model showed moderate discrimination for pathological LNM in NECC when evaluated at the second center (AUC, 0.783). The model combined an intratumoral T1WI-CE score with a 3 mm peritumoral T1WI-CE plus DWI score. Its external AUC did not differ significantly from that of the clinical/MRI comparator, and adding clinical variables did not demonstrate further improvement in external discrimination. Whether these MRI features provide clinically useful information beyond conventional clinical and MRI assessment remains uncertain.

NECC is associated with early lymphatic and hematogenous spread and poorer prognosis than common cervical cancer histologies, with limited prospective management evidence [1,6,8]. Nodal assessment informs staging, prognosis, radiotherapy field design, surgical nodal evaluation, systemic relapse risk assessment, and multidisciplinary planning. Conventional MRI relies on nodal size and morphology and may miss micrometastases or metastases in normal-sized nodes [12,13,35]. Meta-analyses report moderate sensitivity for cervical cancer nodal metastasis [14]. Neither fusion model demonstrated superiority over conventional MRI.

Most cervical cancer radiomics studies of LNM prediction have predominantly included patients with squamous cell carcinoma or adenocarcinoma [21]. MRI-based radiomics models and nomograms have been developed in these populations [35,36], while meta-analyses have summarized their diagnostic performance [20,22]. Differences in biology, metastatic behavior, systemic relapse risk, and treatment limit extrapolation to NECC. Variation in histology, stage, MRI protocols, segmentation, endpoints, and validation also limits cross-study AUC comparisons. Evaluation at a second center therefore provides an initial assessment of performance beyond model development.

The clinical/MRI model showed moderate external discrimination but limited sensitivity. Maximum tumor diameter, MRI-assessed uterine corpus involvement, and retrospectively reconstructed FIGO 2009 stage primarily describe the anatomical extent of disease. The radiomics-only model had a numerically higher external AUC, but the confidence intervals were wide and the difference was not statistically significant. We therefore cannot conclude from this cohort that radiomics provides independent predictive value beyond these conventional predictors.

The final intratumoral signature used T1WI-CE, while the peritumoral signature combined T1WI-CE and DWI. Their selection does not establish superiority over T2WI, which remains important for delineating tumor morphology and local extent because of its high soft-tissue contrast [12,37]. DWI and contrast-enhanced imaging provide additional information about water diffusion and enhancement patterns, respectively. Echo-planar DWI is susceptible to geometric distortion, while contrast-enhanced measurements are affected by acquisition variability and limited standardization [38,39]. Whether these combinations recur in other cohorts and what histologic or biological characteristics they reflect require further validation.

The model based on the 3 mm peritumoral T1WI-CE plus DWI signature had a numerically higher external AUC than the intratumoral model. Both scores were retained in the selected radiomics-only fusion model, although the incremental value of peritumoral information remains uncertain. Previous early-stage cervical cancer studies reported improved LNM prediction after adding peritumoral features [40]. These may reflect stromal or invasive-front tissue characteristics [40,41], but their histopathologic basis was not established here. The MRI-defined 3 mm annulus does not directly measure the histologic invasive front or microscopic lymphovascular invasion.

The radiomics-only model had numerically higher sensitivity than conventional MRI, but the paired comparison was not significant. Its current accuracy, imperfect calibration, and small external cohort do not establish clinical usefulness or improved radiologist performance. If prospectively validated, it could support decisions about further nodal evaluation when MRI findings are equivocal, before multidisciplinary treatment planning. It should not currently guide treatment decisions or replace pathological nodal assessment.

Clinical use of predicted probabilities requires adequate calibration as well as discrimination [42,43]. External calibration was poorer than development calibration: the jointly estimated intercept was −0.346 and the slope was 0.672, both with wide confidence intervals. This joint intercept is not a measure of calibration-in-the-large. Decision curve analysis favored the radiomics-only model over treat-all and treat-none at several thresholds under specified assumptions, without establishing improved patient outcomes [33,44]. Clinical application requires recalibration, justified thresholds, and prospective evaluation of effects on decision making.

Rad-score_Peritumor had a larger mean absolute SHAP contribution to external predictions than Rad-score_Tumor. This ranking describes contributions to the model’s probability output under the development-cohort reference distribution, not biological importance or causality. The scores were correlated, so marginal SHAP explanations cannot establish independent predictor effects. Reproduction requires the stored feature scaling, score formulas, and fitted KNN classifier.

Previous multiomic and single-cell studies have characterized molecular and cellular heterogeneity in small-cell neuroendocrine carcinoma of the cervix [26,45,46]. The exploratory snRNA-seq analysis did not identify imaging–transcriptomic associations that survived correction for multiple testing. The selected correlations in the Supplementary Material therefore remain descriptive observations from a small cohort. They do not establish a biological mechanism of LNM or explain why the radiomics model predicts nodal status.

This study addressed NECC, which is underrepresented in radiomics research. Feature, ROI, and sequence selection, classifier selection and tuning, and model fitting were repeated within nested cross-validation training partitions. Final models were locked before second-center evaluation and compared with the clinical/MRI model. Calibration and decision curves complemented AUC by assessing probability reliability and potential clinical utility [42,47].

Restriction to untreated, histologically confirmed NECC with pathological nodal assessment limits generalizability. Negative selective sampling or excision cannot exclude metastasis in unsampled nodes and may misclassify nodal status. The systematic lymphadenectomy subgroup provided a descriptive assessment with a more consistent nodal-assessment procedure, but included only 29 external patients, 8 with LNM. Its imprecise estimates neither establish superiority nor resolve potential reference-standard bias in the full cohort. Development subgroup results represent apparent performance.

The retrospective design and rarity of NECC constrained the overall sample size, particularly in the external cohort, where small numbers of patients with and without LNM produced wide confidence intervals. The higher proportion of image-quality exclusions at Center 2 may also have affected cohort composition. Model performance in patients with poorer-quality MRI remains uncertain.

Nested cross-validation evaluated the revised selection procedure, not the final fitted model, and cannot capture uncertainty from earlier analytical decisions. Both cohorts had already been examined in the original analysis, so this reanalysis did not evaluate a previously untouched dataset. Development performance requires caution, and external findings remain preliminary. Larger multicenter cohorts are needed to assess model stability, recalibrate probabilities, and establish actionable thresholds.

Scanner, field-strength, and protocol variation may persist despite preprocessing, registration quality control, intensity normalization, and ICC-based filtering. ComBat was not applied, and robustness to alternative harmonization remains unknown. The occupancy audit covered only a 20-patient intratumoral technical subset; three retained features came from image types with low occupancy. This does not itself invalidate those features, but robustness across discretization settings and ROI definitions requires further evaluation. Manual delineation and annulus construction add measurement variability. Acquisition with 6 mm slices and interslice gaps limited delineation of the 3 mm annulus despite resampling to 1 mm isotropic voxels. Resampling cannot restore missing detail, and partial-volume and interpolation effects may influence peritumoral features. The annulus is therefore an image-defined sampling region, not a precisely resolved tissue layer or histologic invasive front.

The transcriptomic analysis included only eight tumors, with some subtype-level pathway means based on few nuclei. Broad screening and post hoc selection limit interpretation. Tumor snRNA-seq cannot localize transcriptional programs to the 3 mm peritumoral imaging region. Independent cohorts with paired imaging and spatially characterized tissue are needed to assess the regional Rad-scores’ biological correlates.

In this two-center NECC cohort, the selected radiomics-only model combined intratumoral T1WI-CE and 3 mm peritumoral T1WI-CE + DWI scores and showed moderate discrimination at the second center. Adding clinical variables did not demonstrate further improvement in external discrimination, and calibration remained imperfect. Larger multicenter cohorts and prospective studies are needed to determine whether the model provides clinically useful information beyond conventional nodal assessment.

## Figures and Tables

**Table 1:** Baseline characteristics of the development and external validation cohorts.

Characteristic	Overall Cohort (*n* = 133)	Development Cohort (*n* = 93)	External Validation Cohort (*n* = 40)	SMD	*p* Value
Age, years	50.26 ± 10.16	50.57 ± 10.21	49.55 ± 10.16	0.100	0.598
Maximum tumor diameter, cm	4.48 ± 3.05	4.41 ± 2.96	4.64 ± 3.30	0.071	0.897
Pathological LNM-positive	54 (40.6)	38 (40.9)	16 (40.0)	0.018	0.926
FIGO 2009 stage				0.326	0.117
Stage I	18 (13.5)	10 (10.8)	8 (20.0)		
Stage II	74 (55.6)	54 (58.1)	20 (50.0)		
Stage III	20 (15.0)	17 (18.3)	3 (7.5)		
Stage IV	21 (15.8)	12 (12.9)	9 (22.5)		
Histologic subtype				0.136	0.429
Small-cell neuroendocrine carcinoma	126 (94.7)	89 (95.7)	37 (92.5)		
Large-cell neuroendocrine carcinoma	7 (5.3)	4 (4.3)	3 (7.5)		
Biopsy-reported mixed histology				0.033	0.864
No	85 (63.9)	59 (63.4)	26 (65.0)		
Yes	48 (36.1)	34 (36.6)	14 (35.0)		
Parametrial involvement (MRI)	68 (51.1)	48 (51.6)	20 (50.0)	0.032	0.865
Vaginal involvement (MRI)	98 (73.7)	70 (75.3)	28 (70.0)	0.118	0.527
Uterine corpus involvement (MRI)	40 (30.1)	29 (31.2)	11 (27.5)	0.081	0.671
Vesicouterine space involvement (MRI)	33 (24.8)	22 (23.7)	11 (27.5)	0.088	0.638
Rectouterine space involvement (MRI)	38 (28.6)	28 (30.1)	10 (25.0)	0.115	0.550
Systemic immune-inflammation index	709.10 ± 491.49	725.44 ± 481.71	671.10 ± 517.78	0.109	0.296
Neutrophil-to-lymphocyte ratio	2.48 ± 1.39	2.51 ± 1.39	2.43 ± 1.41	0.058	0.645
Platelet-to-lymphocyte ratio	154.46 ± 60.42	157.92 ± 58.81	146.43 ± 64.07	0.187	0.246
Prognostic nutritional index	50.10 ± 4.90	49.93 ± 4.18	50.49 ± 6.31	0.104	0.740

Note: Values are mean ± standard deviation or *n* (%). SMDs and *p* values compare the development and external validation cohorts; tests are specified in Section 2.8. For variables with more than two categories, SMD is the maximum absolute category-specific SMD. FIGO 2009 stage was reconstructed using pretreatment information only. Vesicouterine and rectouterine space involvement on MRI alone does not establish bladder or rectal mucosal invasion or FIGO stage IVA. Abbreviations: FIGO, International Federation of Gynecology and Obstetrics; LNM, lymph node metastasis; MRI, magnetic resonance imaging; SMD, standardized mean difference.

**Table 2:** Diagnostic performance of clinical/MRI, radiomics, and clinicoradiomics models for predicting pathological lymph node metastasis.

Model	Cohort	AUC (95% CI)	Accuracy	Sensitivity	Specificity	PPV	NPV	Balanced Accuracy	DeLong *p* vs. Clinical/MRI Model
Clinical/MRI model	Development cohort (*n* = 93)	0.749 (0.647–0.851)	0.720	0.474	0.891	0.750	0.710	0.682	—
Clinical/MRI model	External validation cohort (*n* = 40)	0.727 (0.561–0.893)	0.650	0.438	0.792	0.583	0.679	0.615	Reference
Intratumoral T1WI-CE radiomics signature	Development cohort (*n* = 93)	0.859 (0.779–0.938)	0.796	0.658	0.891	0.806	0.790	0.774	—
Intratumoral T1WI-CE radiomics signature	External validation cohort (*n* = 40)	0.732 (0.572–0.892)	0.675	0.625	0.708	0.588	0.739	0.667	0.935
3 mm peritumoral T1WI-CE + DWI radiomics signature	Development cohort (*n* = 93)	0.862 (0.783–0.940)	0.806	0.658	0.909	0.833	0.794	0.783	—
3 mm peritumoral T1WI-CE + DWI radiomics signature	External validation cohort (*n* = 40)	0.807 (0.668–0.946)	0.775	0.688	0.833	0.733	0.800	0.760	0.142
Radiomics-only model	Development cohort (*n* = 93)	0.876 (0.801–0.950)	0.828	0.763	0.873	0.806	0.842	0.818	—
Radiomics-only model	External validation cohort (*n* = 40)	0.783 (0.630–0.935)	0.800	0.813	0.792	0.722	0.864	0.802	0.333
Clinicoradiomics model	Development cohort (*n* = 93)	0.867 (0.790–0.943)	0.796	0.553	0.964	0.913	0.757	0.758	—
Clinicoradiomics model	External validation cohort (*n* = 40)	0.767 (0.619–0.915)	0.700	0.625	0.750	0.625	0.750	0.688	0.492

Note: Development results represent apparent performance. AUC confidence intervals were calculated using the DeLong method. Classification metrics used a fixed probability threshold of 0.50. Balanced accuracy is the mean of sensitivity and specificity. Reported *p* values are unadjusted, two-sided DeLong comparisons against the clinical/MRI model in the external cohort. No comparison was significant after Holm correction across all 10 pairwise model comparisons. Abbreviations: AUC, area under the receiver operating characteristic curve; CI, confidence interval; DWI, diffusion-weighted imaging; MRI, magnetic resonance imaging; NPV, negative predictive value; PPV, positive predictive value; T1WI-CE, contrast-enhanced T1-weighted imaging.

## Data Availability

The fitted prediction models, full-precision transformations and score formulas, patient-level reference inputs and outputs, and prediction-reproduction script are provided in S5_Model_Files.zip (File S4). Gray-level occupancy records for the 20-patient intratumoral technical QC subset, summary statistics, and mappings to the final signature features are provided in Radiomics_QC_Source_Data.zip (File S5). De-identified patient-level regional Rad-scores, cell proportions, pathway means, barcode-matching counts, complete correlation results, and a verification script for the exploratory transcriptomic analysis are provided in Transcriptomic_Source_Data.zip (File S2). The transcriptomic archive permits reproduction of the reported patient-level correlations and does not constitute a release of the raw sequencing data. The analysis code used for PyRadiomics preprocessing, feature selection, model construction, and statistical analysis is available from the corresponding author upon reasonable request.

## Supplementary Materials

The following supporting information can be downloaded at: https://www.mdpi.com/article/10.3390/curroncol33100625/s1, Figure S1: Regional Rad-score distributions and apparent development performance; Figure S2: Apparent development discrimination, calibration, and decision curve analysis (*n* = 93); Figure S3: Cellular atlas and major cell-type composition of the matched FFPE snRNA-seq subset; Figure S4: Cell-subtype annotation in the matched snRNA-seq subset; Figure S5: Cell-type markers, subtype composition, and exploratory imaging–transcriptomic associations; Table S1: Candidate and excluded pretreatment predictors and coding rules; Table S2: MRI acquisition parameters and scanner distribution by center; Table S3: Radiomics preprocessing and feature extraction settings; Table S4: Feature-count trajectory from initial extraction to final selection; Table S5A: Raw-feature score construction formulas; Table S5B: Final classifier specifications and clinical logistic coefficients; Table S5C: Final classifier input transformations and coding; Table S5D: Radiomic-feature scaling and coefficients for the two final RadScores; Table S6: Databases, reference files, and software used for exploratory FFPE snRNA-seq analysis; Table S7: Machine-learning classifier benchmarking; Table S8: Confusion matrices at the fixed probability threshold of 0.50; Table S9: Cell-level metadata for single-cell RNA-seq cells; Table S10: Patient-level counts and proportions of major cell types; Table S11A: Top differentially expressed markers for each annotated B cell subpopulation; Table S11B: Top differentially expressed markers for each annotated T cell subpopulation; Table S11C: Top differentially expressed markers for each annotated epithelial cell subpopulation; Table S11D: Top differentially expressed markers for each annotated fibroblast subpopulation; Table S11E: Top differentially expressed markers for each annotated macrophage subpopulation; Table S12A: Patient-level proportional distributions of B cell subsets; Table S12B: Patient-level proportional distributions of T cell subsets; Table S12C: Patient-level proportional distributions of epithelial cell subsets; Table S12D: Patient-level proportional distributions of fibroblast subsets; Table S12E: Patient-level proportional distributions of macrophage subsets; Table S13: Selected exploratory correlations between regional Rad-scores and patient-level pathway activity; Table S14: Conventional MRI nodal assessment versus the locked fusion models; Table S15A: Nodal-assessment categories and pathological outcomes by center; Table S15B: Locked radiomics-only model and conventional MRI in the systematic-lymphadenectomy subgroup; Table S15C: Patient-level clinical data; Table S16: Model selection in each outer fold of nested cross-validation; File S1: Supplementary Methods (Supplementary Methods.docx); File S2: Patient-level source data and verification materials for the exploratory transcriptomic analysis (Transcriptomic_Source_Data.zip); File S3: MRI feature-extraction settings (MRI_feature.yaml); File S4: Fitted prediction models, full-precision transformations and score formulas, patient-level reference inputs and outputs, software requirements, and the prediction-reproduction script (S5_Model_Files.zip); File S5: Gray-level occupancy records for the 20-patient intratumoral technical QC subset, sequence-by-image-type summaries, and mapping to the final signature features (Radiomics_QC_Source_Data.zip).
