# Supplementary material for: MRI-Based Radiomics Model for Pretreatment Prediction of Pathological Lymph Node Metastasis in Neuroendocrine Carcinoma of the Cervix: Dual-Center Development and External Validation
Source: Curr Oncol. 2026 Oct 16;33(10):625. doi: 10.3390/curroncol33100625 (PMC13703500; doi:10.3390/curroncol33100625)
Supplement: Supplementary file 1 [file curroncol-33-00625-s001.zip › Supplementary Figure.pdf]

Supplementary Figure

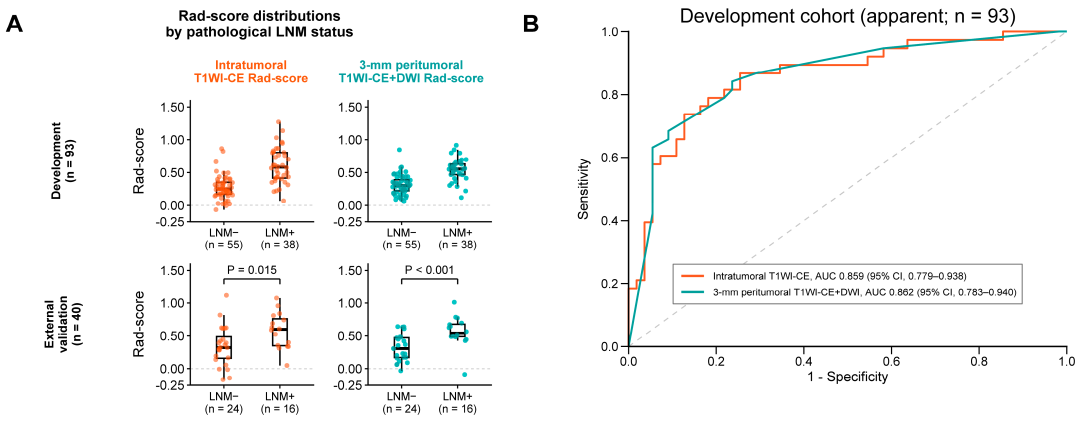

Supplementary Figure S1. Regional Rad-score distributions and apparent development performance. (A) Intratumoral T1WI-CE and 3-mm peritumoral T1WI-CE+DWI Rad-score distributions by pathological LNM status in the development (n = 93; upper row) and external validation (n = 40; lower row) cohorts. Points represent patients; boxes show medians and interquartile ranges, with whiskers extending to observations within 1.5 interquartile ranges. External-cohort P values are from two-sided Wilcoxon rank-sum tests without multiplicity adjustment. (B) Apparent development ROC curves for the intratumoral SVM and peritumoral KNN classifiers, with AUCs and DeLong 95% confidence intervals. Orange and teal denote intratumoral and peritumoral signatures, respectively. These development estimates are not nested out-of-fold performance. T1WI-CE, contrast-enhanced T1-weighted imaging; DWI, diffusion-weighted imaging; LNM, lymph node metastasis; SVM, support vector machine; KNN, k-nearest neighbors; ROC, receiver operating characteristic; AUC, area under the ROC curve.

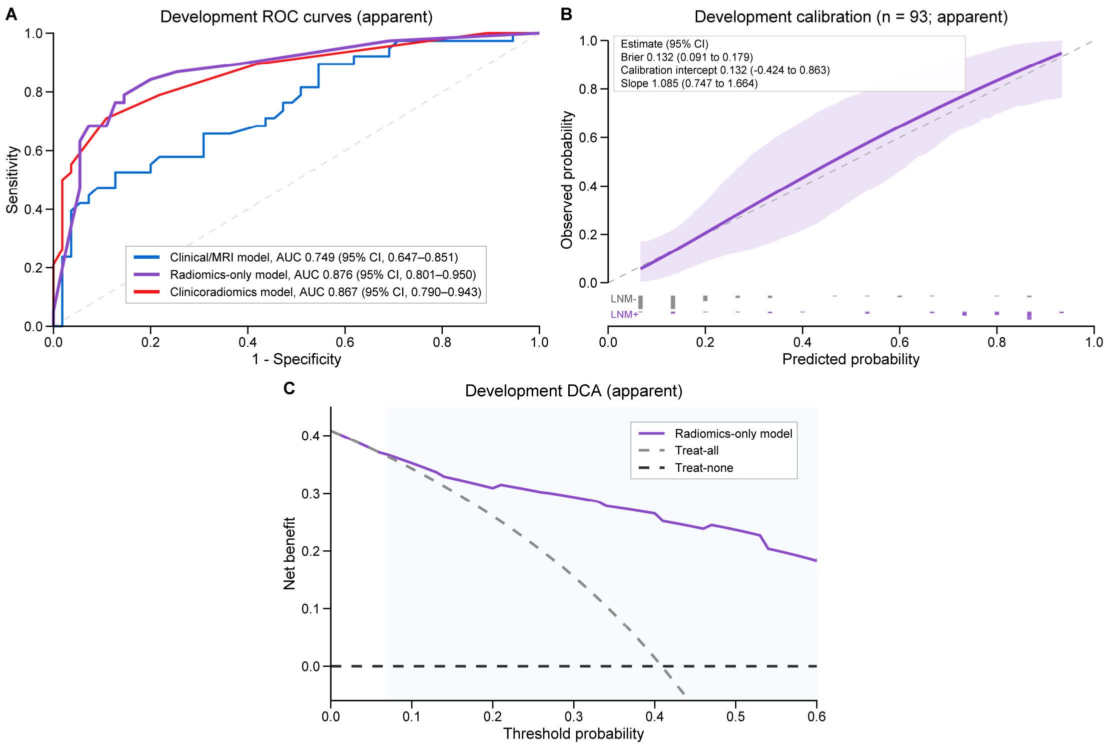

Supplementary Figure S2. Apparent development discrimination, calibration, and decision curve analysis (n = 93). (A) ROC curves for the clinical/MRI, radiomics-only fusion, and clinicoradiomics models, with AUCs and DeLong 95% confidence intervals. (B) Calibration of the radiomics-only KNN fusion model. The curve uses logistic regression with a three-knot restricted cubic spline of logit-transformed predicted probability. The band indicates pointwise 95% confidence intervals from 1,000 patient bootstrap samples, conditional on the fitted model. The dashed diagonal indicates ideal calibration; rugs show individual predictions by LNM status. The inset reports the Brier score and jointly estimated calibration intercept and slope with bootstrap 95% confidence intervals. (C) Decision curves for the radiomics-only model versus treat-all and treat-none. Shading marks evaluated thresholds ⩾0.05 where observed model net benefit exceeds both references. All development results are apparent, without optimism correction, and are distinct from nested out-of-fold estimates. ROC, receiver operating characteristic; AUC, area under the ROC curve; KNN, k-nearest neighbors; LNM, lymph node metastasis.

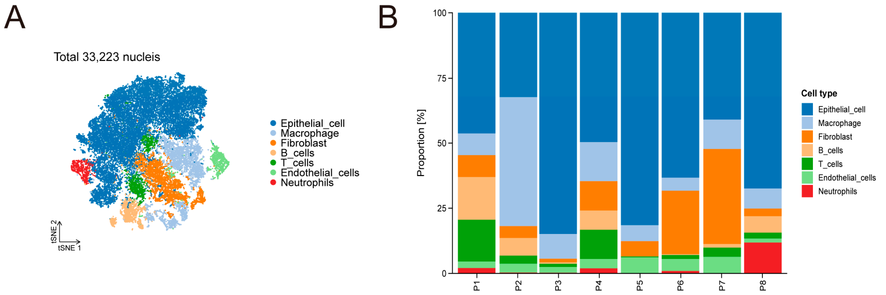

Supplementary Figure S3. Cellular atlas and major cell-type composition of the matched FFPE snRNA-seq subset. (A) t-SNE visualization of seven major cell types using 33,223 randomly downsampled nuclei, approximately 30% of the 110,829 nuclei retained after quality control. The displayed count refers to the visualization subset. (B) Patient-level proportions of the seven major cell types in eight patients (P1–P8). Consistent colors identify the major cell types across panels. FFPE, formalin-fixed paraffin-embedded; snRNA-seq, single-nucleus RNA sequencing; t-SNE, t-distributed stochastic neighbor embedding.

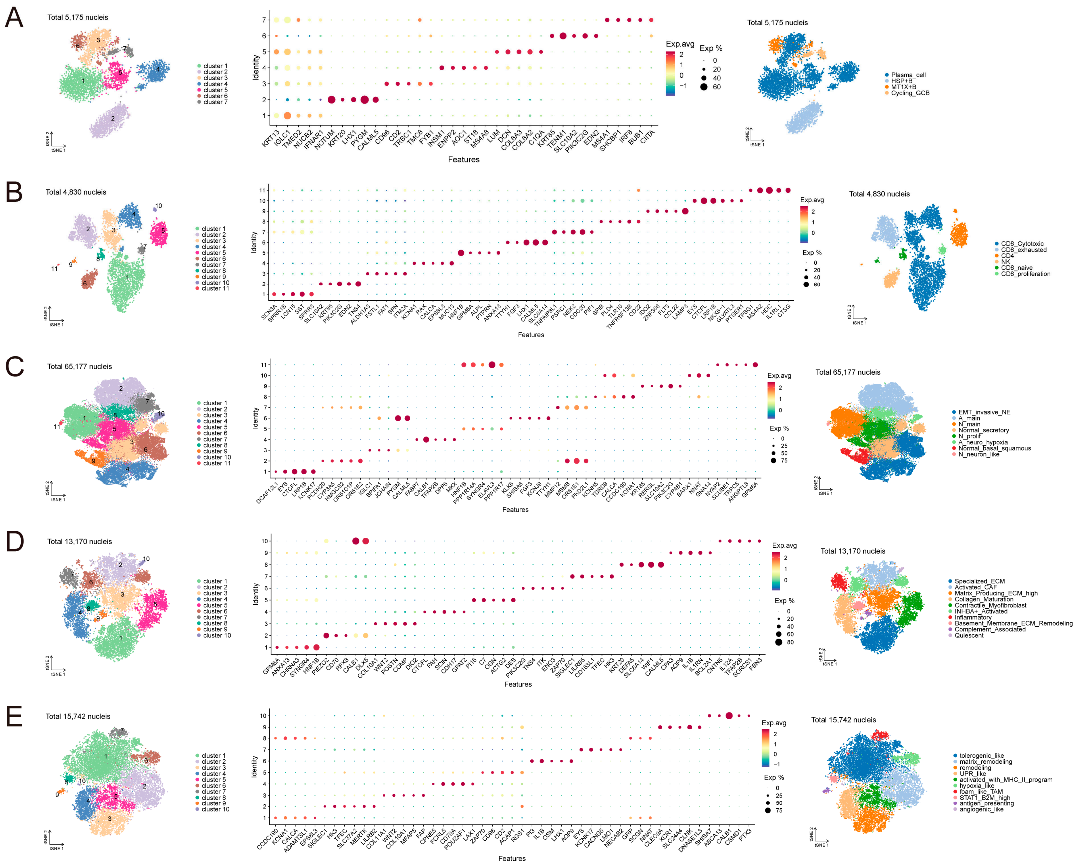

Supplementary Figure S4. Cell-subtype annotation in the matched snRNA-seq subset. Panels show

(A) B cells (5,175 nuclei), (B) T cells (4,830 nuclei), (C) epithelial cells (65,177 nuclei), (D) fibroblasts (13,170 nuclei), and (E) macrophages (15,742 nuclei). Within each panel, the left t-SNE plot shows unsupervised clusters, the middle dot plot shows marker-gene expression across clusters, and the right t-SNE plot shows annotated subtypes. Dot size indicates the percentage of nuclei expressing each gene, and color indicates scaled average expression. Counts refer to nuclei in each major cell class; the biological cohort comprised eight patients. snRNA-seq, single-nucleus RNA sequencing; t-SNE, t-distributed stochastic neighbor embedding.

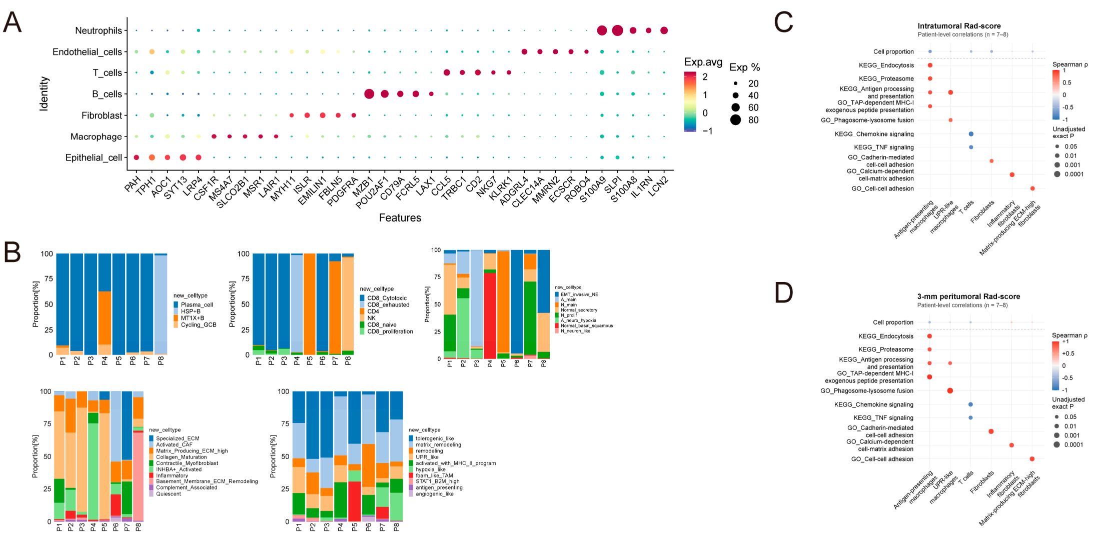

Supplementary Figure S5. Cell-type markers, subtype composition, and exploratory imaging–transcriptomic associations. (A) Representative canonical marker expression for the seven major cell types. Dot size indicates the percentage of nuclei expressing each gene, and color indicates scaled average expression. (B) Patient-level subtype composition within B cells, T cells, epithelial cells, fibroblasts, and macrophages, shown from left to right in the upper row and then the lower row. (C, D) Patient-level correlations with the intratumoral T1WI-CE (C) and 3-mm peritumoral T1WI-CE+DWI (D) Rad-scores. The 11 previously selected cell–pathway pairs are retained as post hoc descriptive examples; all had unadjusted two-sided exact permutation P < 0.05 with concordant directions for both scores. Corresponding cell proportions are shown irrespective of P value. Color indicates Spearman's rho (red, positive; blue, negative), and circle area is proportional to −3 × log10(P), using common scales across panels C and D. Pathway correlations for antigen-presenting macrophages used seven patients because P5 lacked the corresponding pathway measurements; all other displayed correlations used eight

patients. No association survived Benjamini–Hochberg correction across all eligible tests within each regional Rad-score. Blank positions indicate combinations not selected for display. These observations do not establish a biological mechanism of LNM. Supplementary Table S13 and Transcriptomic_Source_Data.zip provide association statistics and patient-level measurements. T1WI-CE, contrast-enhanced T1-weighted imaging; DWI, diffusion-weighted imaging; LNM, lymph node metastasis; KEGG, Kyoto Encyclopedia of Genes and Genomes; GO, Gene Ontology.
