# Supplementary material for: MRI-Based Radiomics Model for Pretreatment Prediction of Pathological Lymph Node Metastasis in Neuroendocrine Carcinoma of the Cervix: Dual-Center Development and External Validation
Source: Curr Oncol. 2026 Oct 16;33(10):625. doi: 10.3390/curroncol33100625 (PMC13703500; doi:10.3390/curroncol33100625)
Supplement: Supplementary file 1 [file curroncol-33-00625-s001.zip › Supplementary Methods.pdf]

Supplementary Methods

Nodal assessment and treatment context

The retrospective cohort comprised patients with histologically confirmed neuroendocrine carcinoma of the cervix (NECC), pretreatment MRI, and pathological nodal assessment. Nodal tissue was obtained by lymphadenectomy or sampling, with or without definitive primary tumor resection. Both MRI and the nodal tissue used to establish the endpoint were obtained before antitumor treatment. Patients undergoing surgery after neoadjuvant chemotherapy were excluded.

Most patients with FIGO 2009 stage I or II disease, including selected patients with stage IIB disease, underwent radical hysterectomy with pelvic lymphadenectomy. Among patients with stage III disease, some underwent radical hysterectomy with pelvic lymphadenectomy after individualized assessment, whereas others underwent lymph node excision or biopsy without radical hysterectomy. Patients with stage IV disease underwent lymph node excision or biopsy; none underwent radical hysterectomy or systematic lymphadenectomy. Nodal assessment involved pelvic and/or para-aortic nodes, and some enlarged nodes were removed with therapeutic intent.

Both centers used multimodal treatment. Systemic therapy evolved during the accrual period. Platinum-based combinations containing etoposide or paclitaxel were used, with cisplatin plus etoposide becoming the preferred regimen after the introduction of dedicated NCCN recommendations for cervical small-cell neuroendocrine carcinoma. Radical hysterectomy was generally followed by chemotherapy or chemoradiotherapy. Locally advanced disease not managed by primary tumor resection was generally treated with definitive chemoradiotherapy and brachytherapy, while metastatic disease was managed primarily with systemic therapy. The endpoint was pathological lymph node metastasis (LNM), defined as metastatic involvement of at least one resected or sampled node. A negative label meant that no metastasis was detected in the examined nodes, so the reference standard was limited to the tissue obtained during each patient's nodal assessment. Among patients with negative findings after selective nodal excision or biopsy, seven had FIGO 2009 stage III disease and seven had stage IV disease. The median number of examined nodes was 5 in each group, with ranges of 3 – 8 and 3 – 7, respectively. The study evaluated pretreatment nodal-risk prediction in this histologically assessed cohort.

MRI interpretation criteria and pretreatment predictor coding

Two radiologists with 11 and 20 years of experience in pelvic MRI reviewed all pretreatment examinations while blinded to pathological LNM status and clinical outcomes. They recorded MRI-assessed signs of local invasion before model development and resolved disagreements by consensus.

Parametrial involvement was defined by disruption or loss of the low-signal cervical stromal ring on T2WI, irregular tumor extension beyond the cervical stroma into parametrial fat, or nodular infiltration along parametrial soft-tissue planes. Vaginal involvement required direct tumor extension into the vaginal wall or lumen on T2WI and/or contrast-enhanced T1WI, with loss of the normal cervical-vaginal interface. Uterine corpus involvement was continuous tumor extension from the cervix into the lower uterine segment, endometrial cavity, or myometrium of the uterine corpus. Vesicouterine space involvement was defined by tumor-associated obliteration, nodular infiltration, or abnormal soft-tissue extension into the fat plane between the anterior cervix/uterus and posterior bladder wall. The same findings in the posterior cul-de-sac or fat plane between the cervix/uterus and rectum defined rectouterine space involvement.

MRI-suspected nodal metastasis was recorded as a conventional imaging comparator. Assessment used routine pelvic MRI criteria: enlarged short-axis diameter, round morphology, irregular contour, internal necrosis, heterogeneous enhancement, or restricted diffusion. The final consensus assessment was a binary variable evaluated against pathological LNM status. It was excluded from the candidate predictors and compared with the locked radiomics-only fusion and clinicoradiomics models in both cohorts.

Inflammation and nutrition indices were calculated from peripheral blood tests obtained before any antitumor treatment. Systemic immune-inflammation index (SII) was calculated as platelet count × neutrophil count / lymphocyte count. Neutrophil-to-lymphocyte ratio (NLR) was calculated as neutrophil count / lymphocyte count. Platelet-to-lymphocyte ratio (PLR) was calculated as platelet count / lymphocyte count. Prognostic nutritional index (PNI) was calculated as serum albumin (g/L) + 5 × lymphocyte count (10^9/L). SII, NLR, and PLR had been log10-transformed before inclusion in the modeling dataset; no further logarithmic transformation was applied during the revised analysis.

Clinical-variable selection in the revised clinicoradiomics analysis considered thirteen pretreatment variables: five MRI-assessed invasion signs, age, maximum tumor diameter, log10 SII, log10 NLR, log10 PLR, PNI, biopsy-reported mixed histology, and FIGO 2009 stage. The invasion signs were parametrial, vaginal, uterine corpus, vesicouterine space, and rectouterine space involvement. Invasion signs and mixed histology were coded as 0 for absence and 1 for presence. FIGO stage was coded as 0 for IA–IIA2 and 1 for IIB–IVB, with 0 as the reference category. Maximum tumor diameter was expressed in centimeters. Supplementary Table S1 summarizes predictor definitions, coding rules, and excluded variables.

Supplementary MRI acquisition parameters

MRI examinations were performed on 1.5-T or 3.0-T scanners, including GE Signa 1.5T EXCITE III HD, GE Discovery 750w 3.0T, and Siemens Prisma 3.0T systems, using phased-array body coils. Supplementary Table S2 summarizes the recorded scanner manufacturer/model, field strength, sequence, orientation, repetition time, echo time, flip angle when applicable, field of view, acquisition matrix, slice thickness, interslice gap, number of slices, acquisition time, and number of excitations, together with the scanner distribution by center. Its footnote specifies the DWI b value used for radiomics extraction. All patients at both centers received gadoterate meglumine at a dose of 0.2 mL/kg body weight. Acquisition of the T1WI-CE images used for radiomics began approximately 2 min after the start of contrast injection.

The radiomics workflow used source axial T2WI, axial DWI with b = 800 s/mm², and T1WI-CE images. Apparent diffusion coefficient maps were available for routine clinical interpretation of nodal status but were not included in either the primary or supplementary radiomics models because the prespecified feature-extraction workflow was restricted to source axial T2WI, b = 800 s/mm² DWI, and T1WI-CE images.

Image preprocessing and registration quality control

All DICOM images were imported into 3D Slicer version 5.8.1 and converted to NIfTI format. Axial T2WI served as the anatomical reference sequence. DWI and T1WI-CE were rigidly registered to axial T2WI using 3D Slicer. Registration quality was visually inspected before feature extraction. Cases with gross

misregistration were corrected manually when feasible; cases with non-correctable registration failure were excluded and reported in the study flow diagram.

Images were resampled to isotropic 1.0-mm voxels using linear interpolation, and segmentation masks were resampled using nearest-neighbor interpolation. N4 bias-field correction was applied to the resampled images, followed by z-score normalization using the whole-volume mean and standard deviation. The revised feature-extraction run used these previously normalized images with PyRadiomics internal normalization disabled. Gray-level discretization used a fixed bin width of 0.05 on the normalized intensity scale. No post-extraction feature harmonization, including ComBat, was applied.

Radiomics feature extraction settings

Radiomics features were extracted using PyRadiomics version 3.1.0, with feature definitions following the Image Biomarker Standardisation Initiative where applicable. The revised YAML configuration specified binWidth = 0.05, normalize = False, resampledPixelSpacing = [1, 1, 1], and interpolator = sitkBSpline. Extraction used three-dimensional, segment-based features, and image–mask geometry was checked before extraction. Extractor-level resampling remained enabled. Image interpolation is bypassed when the input spacing matches the target, although nearest-neighbor mask resampling and cropping may still occur. Additional diagnostic fields were disabled in the exported feature output (additionalInfo = False). Supplementary Table S3 and MRI_feature.yaml provide the extraction settings.

Features were extracted from original and filtered images. Filtered images included Laplacian of Gaussian filters with σ = 1.0, 3.0, and 5.0 mm, wavelet decompositions, and square, square-root, logarithm, and exponential intensity transforms. Extracted feature classes included shape, first-order statistics, gray-level co-occurrence matrix, gray-level run length matrix, gray-level size zone matrix, gray-level dependence matrix, and neighboring gray tone difference matrix features. Shape descriptors were computed from the ROI masks within each image–mask extraction. First-order and texture descriptors were extracted from the original and filtered images for each MRI sequence.

Gray-level occupancy was assessed in a technical QC subset of 20 patients using the second observer's intratumoral masks and the preprocessed images shared by the observers. At binWidth = 0.05, the number of distinct occupied discretized gray levels was recorded for each patient, MRI sequence, and

image type. Low occupancy was operationally defined as two or fewer occupied levels within the ROI. This definition was used as a descriptive QC flag and was not treated as evidence that an individual feature was invalid. The three sequences and 16 image types yielded 960 records. Fifty-eight records (6.04%) were flagged, all with exactly two occupied levels and all in wavelet-filtered images. No original-image record was flagged (0/60). Counts were 24/320 for T1WI-CE, 2/320 for T2WI, and 32/320 for DWI. By wavelet subband, counts were 39/60 for HHH, 4/60 for HHL, 9/60 for HLH, and 6/60 for LHH; the other image types had no flagged records.

For the four T1WI-CE wavelet image types contributing intensity or texture features to the final intratumoral score, the median (range) numbers of occupied gray levels across the 20 intratumoral QC cases were 12 (7 – 25) for HHL, 44 (21 – 84) for LHL, 4 (2 – 6) for LHH, and 2 (2 – 4) for HHH. The largest-bin fraction was defined as the proportion of ROI voxels assigned to the most populated discretized gray-level bin. Its median (range) was 39.03% (32.30 – 48.93%), 13.51% (7.52 – 20.51%), 50.31% (49.87 – 52.25%), and 50.11% (50.00 – 51.02%), respectively. These summaries apply only to the 20 second-observer intratumoral masks at binWidth = 0.05. Peritumoral occupancy was not assessed.

The final intratumoral signature contains three features from affected T1WI-CE image types: wavelet-LHH GLCM MaximumProbability and wavelet-LHH NGTDM Busyness (4/20 low-occupancy cases for this image type), and wavelet-HHH GLDM DependenceEntropy (18/20). Each feature had finite, nonconstant values within its corresponding flagged cases. These checks do not establish robustness to discretization or acquisition differences. These features also contribute to the imaging-fusion and clinicoradiomics predictors through the intratumoral score. The audit did not measure occupancy in peritumoral ROIs or throughout the complete modeling cohort. Full QC records, a sequence-by-image-type summary, and the mapping to all nonzero-coefficient signature features are supplied in Radiomics_QC_Source_Data.zip.

Tumor segmentation, peritumoral annulus editing, and selection of peritumoral setting

Primary tumor volumes of interest were manually delineated slice by slice on axial T2WI using 3D Slicer version 5.8.1. After registration to axial T2WI, DWI and T1WI-CE provided additional references for confirming boundaries. The volumes included cystic, necrotic, and hemorrhagic components to capture the whole-tumor imaging phenotype.

Peritumoral annuli were created by morphologically dilating the tumor volume outward by 3 mm and 5 mm and subtracting the original tumor volume. The annuli were then manually edited according to predefined anatomical rules. Areas outside the body, bowel gas, bone, large vessels, bladder lumen, rectal lumen, and obvious non-tumor structures included by automatic dilation were removed. The edited T2WI-based annuli were propagated to the registered DWI and T1WI-CE images where applicable.

Seven non-empty combinations of T1WI-CE, T2WI, and DWI were evaluated for the tumor, the 3-mm peritumoral annulus, and the 5-mm annulus, yielding 21 regional imaging configurations. T1 denotes T1WI-CE in the exported analysis files. Regional and sequence selection used development-cohort inner-fold AUCs within the staged procedure described below. The final configurations were intratumoral T1WI-CE and the 3-mm peritumoral annulus with T1WI-CE plus DWI. External-cohort performance did not determine these choices. Supplementary Table S4 reports the feature-selection trajectory for the final development-cohort fits.

Segmentation reproducibility and radiomics feature selection

Interobserver feature reproducibility was assessed using paired segmentations from 20 development-cohort patients. A second reader independently delineated these cases while blinded to pathological LNM status. The revised pipeline used the two-way random-effects, absolute-agreement, single-measurement intraclass correlation coefficient, ICC(A,1), and retained only features with finite values $\geqslant 0.85$. Screening was performed separately for each relevant regional and sequence configuration. During cross-validation, ICCs were recalculated using only repeat-segmented patients in the corresponding training partition, with no contribution from held-out patients. The implemented minimum was five paired cases per training partition; the final full-development fits used all 20 paired cases. Intraobserver ICC was not a selection criterion in the revised pipeline.

Radiomics preprocessing and feature selection were fitted separately within each relevant training partition, including inner-training fits, outer-training refits, and the final full-development fit. Constant features were removed after ICC screening. The remaining features were standardized using training-partition means and population standard deviations, as implemented in StandardScaler. Feature lists, scaling parameters, and score coefficients were then applied unchanged to the corresponding held-out patients.

Redundancy filtering used an absolute Spearman correlation threshold greater than 0.85. Features were assessed in input-column order; a later feature was removed if its correlation with an earlier feature exceeded the threshold. Recursive feature elimination (RFE) then used a 100-tree random forest, removing one feature per iteration until at most 10 remained. LASSO linear regression against the binary LNM outcome generated a continuous Rad-score. Fivefold stratified cross-validation selected the penalty by minimizing mean squared error over 100 logarithmically spaced values from 10^{-6} to 10^{1}. LASSO used a maximum of 20,000 iterations and a tolerance of 10^{-6}. Features with non-zero coefficients contributed to the Rad-score, which was passed to a subsequent classifier and was not itself an LNM probability. Supplementary Table S4 reports the final feature counts; counts could vary across cross-validation folds.

Model development and classifier benchmarking

The revised analysis retained the original center-based split of 93 development patients and 40 external validation patients. Internal validation used stratified nested cross-validation with five outer and five inner folds, shuffled partitions, and random seed 1128. In each outer fold, held-out patients were excluded from the complete staged development procedure. The remaining patients were used for regional and sequence selection, training-partition feature selection, clinical-variable selection, classifier tuning, and model fitting. Pooled outer-fold predictions provided out-of-fold (OOF) performance estimates.

Within each outer training partition, the 21 regional configurations were evaluated using eight classifier families and their parameter grids. Maximum mean inner-fold AUC determined one intratumoral and one peritumoral configuration. Fusion was then evaluated only for this pair, using the two Rad-scores with and without selected clinical variables. The fusion search used the same inner folds and eight classifier families; it did not enumerate all possible intratumoral-peritumoral pairs. Inner-fold fusion scores were

conditional tuning scores, whereas outer-fold predictions evaluated the complete staged selection procedure.

Clinical-variable selection for fusion used LASSO linear regression on the 13 candidates within each training partition. Binary variables used 0 as the reference category. Constant predictors were removed, and the remaining predictors were standardized for LASSO selection. The penalty search used the same fivefold scheme and 100-value range as radiomics score construction. Selected clinical variables entered fusion directly alongside the two Rad-scores. These inputs were standardized using training-partition parameters before classifier fitting. Single-region classifiers received their Rad-score without the additional fusion-input scaling. When no clinical predictors were selected in a training partition, the clinicoradiomics model used the two Rad-scores alone.

OOF performance was reported separately for the intratumoral, peritumoral, radiomics-only fusion, and clinicoradiomics development procedures. A further estimate included selection among eligible model families within each outer training partition. Regional configurations, retained variables, and classifiers could vary across outer folds, so these estimates evaluate development procedures rather than a single fixed final classifier. After internal evaluation, the same procedure was applied to all development patients. Final models were locked before external evaluation, with no external recalibration, threshold modification, or reselection.

The three-predictor clinical/MRI logistic regression model developed in the initial analysis of this study using the 93-patient development cohort was retained as a fixed comparator in the reanalysis. Its predictors were uterine corpus involvement, maximum tumor diameter, and binary FIGO 2009 stage. Existing development and external predictions were imported without refitting or retuning; no corresponding nested OOF predictions were available. This comparator was separate from the newly selected clinical variables used in clinicoradiomics fusion.

The eight classifiers were logistic regression, support vector machine (SVM), k-nearest neighbors (KNN), decision tree, random forest, XGBoost, LightGBM, and Gaussian naive Bayes. Mean inner-fold AUC was the primary selection criterion. For regional and overall model-family selection, exact AUC ties were resolved by a deterministic ordering of profile, classifier, and parameter index. Within fusion searches,

AUC ties within 10^{-12} were resolved by lower mean Brier score, followed by deterministic ordering. No synthetic oversampling was applied. Supplementary Table S7 summarizes classifier benchmarking.

Classifier hyperparameter grids

SVM: C comprised 10 logarithmically spaced values from 2^{-5} to 2^{5}; gamma comprised 50 logarithmically spaced values from 2^{-5} to 2^{1}. Radial basis function, linear, polynomial, and sigmoid kernels were evaluated, with probability estimation enabled.

Logistic regression: penalties were L1 and L2; C values were 0.0001, 0.001, 0.01, 0.1, 1, 10, 100, and 1000. The liblinear and saga solvers were evaluated, with a maximum of 1,000,000 iterations.

KNN: neighbor counts were 3, 5, 7, 9, 11, 13, 15, 17, and 19; weighting was uniform or distance-based. Search algorithms were auto, ball_tree, kd_tree, and brute. Distance metrics were Euclidean, Manhattan, Minkowski, and Chebyshev.

Decision tree: criteria were Gini and entropy; splitters were best and random; maximum depths were 1, 3, 5, 7, and 9. Minimum split sizes were 2, 5, and 10; minimum leaf sizes were 1, 2, and 4; ccp_alpha values were 0.01, 0.05, and 0.1.

Random forest: criteria were Gini and entropy; tree counts were 5, 10, 15, 20, and 25. Maximum depths were 3, 5, 7, 9, 11, 13, 15, 17, and 19.

LightGBM: estimator counts were 5, 10, 15, 20, 25, 50, and 100; learning rates were 0.01, 0.05, and 0.1. Leaf counts were 7, 15, and 31, and max_bin was fixed at 255.

XGBoost: estimator counts were 100, 200, 300, 400, and 500; maximum depths were 3, 4, 5, 6, and 7. Learning rates were 0.01, 0.02, 0.05, and 0.1. Fixed settings included booster = gbtree, objective = binary:logistic, max_leaves = 127, colsample_bytree = 0.6, subsample = 0.7, gamma = 0.1, reg_lambda = 1, and reg_alpha = 1.

Gaussian naive Bayes used the implementation defaults without a hyperparameter search. Other unspecified classifier settings used the defaults of the recorded software versions.

Score construction and locked prediction models

Each Rad-score was calculated from standardized radiomics features using the fitted LASSO intercept and coefficients:

$$\text{Rad-score} = a + \Sigma[j]\ \beta[j] \times (x[j] - \mu[j]) / s[j].$$

Here, x[j] is the extracted feature value; μ[j] and s[j] are its training-partition mean and standard deviation. The equivalent formula on the extracted-feature scale was:

$$\text{Rad-score} = a\_raw + \Sigma[j]\ b[j] \times x[j],$$

where $b[j] = \beta[j] / s[j]$ and $a\_raw = a - \Sigma[j]\ \beta[j] \times \mu[j] / s[j]$.

The final intratumoral T1WI-CE score contained seven non-zero coefficients, and the 3-mm T1WI-CE plus DWI peritumoral score contained four. Supplementary Table S5A provides the Rad-score equations on the extracted-feature scale. Supplementary Table S5B specifies the final downstream classifiers and the clinical/MRI comparator's logistic coefficients and intercept. Supplementary Table S5C provides classifier-input standardization parameters, input order, and clinical coding. Supplementary Table S5D provides radiomic-feature standardization parameters (development-cohort means and scales), together with standardized- and extracted-feature-scale coefficients. The feature scales in S5D and the non-identity classifier-input scales in S5C use population SDs (denominator n). The single-region classifiers use an identity input transformation, as specified in S5C.

The supplementary archive S5_Model_Files.zip contains the four original fitted estimators in the Intratumoral, Peritumoral, Imaging_fusion, and Clinicoradiomics_selected directories; their prediction_pipeline.json specifications; full-precision score formulas and scaling parameters; the clinical/MRI logistic equation in Clinical_existing/clinical_formula_raw_verified.json; the four patient-level input tables in reference_inputs/; and the expected scores and predictions in reference_outputs/. The root-level script reproduce_predictions.py implements frozen inference from extracted features and clinical variables. The command “python reproduce_predictions.py” runs the reference verification without model refitting. Installation and version information are provided in README.md and

requirements-tested.txt, and executed verification records are provided in verified_reference_run/. Reproduction uses full-precision machine-readable parameters, not rounded table entries. The extracted-feature-scale and standardized-feature-scale score equations are equivalent alternatives and must not be combined by standardizing the inputs twice. T1 in the machine-readable files denotes T1WI-CE. Development outputs represent apparent predictions for 93 patients; external-validation outputs cover 40 patients. All 665 reference probabilities were reproduced with a maximum absolute difference of 6.44×10^{-15}, and all classifications at a probability threshold of 0.50 matched. This verification starts from extracted features and does not repeat image preprocessing, feature extraction, or model selection.

The locked intratumoral classifier was an SVM with a sigmoid kernel, C = 6.857951862824587, and gamma = 1.3083559587480234, with probability estimation enabled. The locked peritumoral classifier was KNN with 17 uniformly weighted neighbors and Euclidean distance. The radiomics-only fusion model used KNN with 15 uniformly weighted neighbors and Euclidean distance, applied to the two standardized Rad-scores. This model was selected as the primary model by the development-cohort selection procedure.

The clinicoradiomics model also used KNN with 15 uniformly weighted neighbors and Euclidean distance. Its inputs were the two Rad-scores and seven selected clinical variables. These were parametrial involvement, uterine corpus involvement, vesicouterine space involvement, maximum tumor diameter, log10 SII, PNI, and binary FIGO 2009 stage. All nine inputs were standardized using the final development-cohort means and scales. KNN probabilities were the proportion of LNM-positive training observations among the selected neighbors. The fixed clinical/MRI comparator retained its original three-variable logistic probability function.

Reproducing SVM or KNN probabilities requires the fitted classifiers as well as the Rad-score formulas. Logistic probability coefficients and odds ratios apply only to the clinical/MRI comparator, not to the SVM and KNN models. Supplementary Table S8 reports the classification threshold and confusion matrices.

Model evaluation details

The primary discrimination metric was the area under the receiver operating characteristic curve (AUC). External AUCs were reported with 95% confidence intervals calculated using the DeLong normal approximation and truncated to the interval from 0 to 1. These intervals were conditional on the locked models. Apparent development performance and nested OOF performance were reported separately. Threshold-dependent metrics comprised accuracy, sensitivity, specificity, positive predictive value, negative predictive value, balanced accuracy, and confusion matrices. A predicted probability ⩾0.50 indicated LNM positivity, with the same threshold used for all cohorts and models. Proportion-based classification metrics were reported with 95% Wilson confidence intervals where indicated.

For the retrospective descriptive analysis by nodal-assessment procedure, patients were classified as having undergone systematic lymphadenectomy or selective nodal excision or biopsy only. Category counts and pathological outcomes were summarized separately for the development and external-validation cohorts. The subgroup evaluation was restricted to patients who underwent systematic lymphadenectomy. For each included patient, we used the unchanged probability from the locked radiomics-only KNN fusion model (Imaging_fusion in S5_Model_Files.zip) and the recorded conventional MRI nodal assessment. Model classifications used the fixed probability threshold of 0.50. No model refitting, feature reselection, recalibration, or threshold optimization was performed. Confusion matrices and sensitivity, specificity, and accuracy with two-sided 95% Wilson score confidence intervals were reported separately by center (Supplementary Table S15). These intervals describe binomial uncertainty conditional on the recorded reference standard and fixed predictions; they do not incorporate model-development uncertainty or possible pathological misclassification. Development model results represent apparent performance, not out-of-fold estimates. The subgroup comparisons were descriptive, without paired hypothesis testing.

Patient-matched DeLong tests evaluated all 10 pairwise AUC comparisons among the five distinct reported models in the external cohort. Both unadjusted P values and P values adjusted by the Holm method across these 10 comparisons were reported. The comparisons were exploratory and did not determine model selection. Duplicate export labels for the same locked model were not counted as additional independent models.

Conventional MRI nodal assessments were matched to model predictions by patient identifier and evaluated against the same pathological outcome. Two-sided exact McNemar tests compared MRI with the radiomics-only fusion and clinicoradiomics models: sensitivity among pathology-positive patients, specificity among pathology-negative patients, and accuracy among all patients. These exploratory comparisons were reported without multiplicity adjustment. Development-cohort comparisons used apparent training predictions. Positive and negative predictive values were descriptive and were not included in the paired tests. Supplementary Table S14 reports the MRI comparisons.

Calibration was assessed using the Brier score, smooth calibration curves, and a logistic calibration model. Figure 3B and Supplementary Figure S2B show calibration of the locked radiomics-only KNN model from individual predictions in the external validation and development cohorts, respectively. Smooth curves were fitted by binomial logistic regression with a restricted cubic spline of logit(p), with knots at its cohort-specific 10th, 50th, and 90th percentiles; p denotes predicted LNM probability. Curves were displayed over the observed prediction range with 95% pointwise bootstrap bands, an ideal line, and individual-prediction rugs. No grouped calibration points were displayed. A separate linear logistic recalibration model jointly estimated the intercept and slope from logit(p). The figure label "Calibration intercept" denotes this jointly estimated intercept, not calibration-in-the-large with the slope fixed at 1. Probabilities were clipped to $[10^{-8}, 1 - 10^{-8}]$ only for logit-based diagnostics; original model probabilities were otherwise unchanged.

External Brier scores and calibration parameters were reported with percentile intervals from 1,000 patient-level bootstrap resamples, using random seed 1128. Each resample drew 40 patients with replacement and preserved paired predictions across models. For Figure 3B and Supplementary Figure S2B, 1,000 resamples were used separately within each cohort to estimate pointwise curve bands and intervals for Brier scores and calibration parameters; development resamples contained 93 patients. Spline knots remained fixed at their original cohort values, and calibration regressions were refitted in each resample. Predictive models were neither refitted nor reselected. Intervals used the 2.5th and 97.5th percentiles of valid estimates. Non-estimable calibration fits were excluded, and valid replicate counts were recorded. The intervals quantify uncertainty conditional on the locked models and exclude

model-development uncertainty. Development calibration was apparent and was not corrected for optimism. Hosmer–Lemeshow tests were not used.

Decision curve analysis used locked-model predictions, with apparent development performance and external performance reported separately. Net benefit was calculated over thresholds from 0.01 to 0.99 in increments of 0.01 as $TP/n - (FP/n) \times t/(1 - t)$, where t denotes threshold probability, and compared with treat-all and treat-none strategies. Figure 3C and Supplementary Figure S2C also include threshold zero and display thresholds up to 0.60. The model-directed strategy specified intensified pretreatment nodal evaluation and multidisciplinary review above a selected threshold. No established clinical decision threshold was assumed for NECC. These exploratory analyses did not establish recommended clinical cutoffs.

Visualization and explanation of the final KNN model

Figure 4A shows the locked radiomics-only KNN model over a 151 × 151 grid spanning the two development-cohort Rad-scores, with the stored development scaling parameters applied. The display was restricted to the convex hull of development score pairs and overlaid with all 93 patients by pathological LNM status. No surface smoothing or probability recalibration was applied. The dashed decision boundary was derived from grid indicators of predicted probability $\geqslant 0.50$. Uniformly weighted 15-neighbor probabilities occur in increments of 1/15, so this boundary approximates a class transition at the grid resolution; it is not an exact 0.50 isoprobability contour. The threshold was not newly optimized.

Figure 4B explains all 40 external predictions using exact marginal Shapley values on the positive-class probability scale, without a logit transformation. All 93 development patients formed the empirical background. With T and P denoting the intratumoral and peritumoral inputs, respectively, $v(\emptyset)$ was the mean prediction over background patients; $v(T)$ and $v(P)$ fixed the corresponding score at the explained patient's value and averaged predictions over background values of the other score; $v(T,P)$ was the patient's prediction. The four coalitions were enumerated, giving $\varphi(T) = 0.5 \times [v(T) - v(\emptyset) + v(T,P) - v(P)]$ and $\varphi(P) = 0.5 \times [v(P) - v(\emptyset) + v(T,P) - v(T)]$. Prediction additivity was checked. Rows were ordered by mean absolute SHAP value, with feature-value colors scaled separately for each Rad-score. Marginal replacement can produce combinations outside the observed joint distribution when scores are

correlated; these attributions describe the fitted model under the specified background, not independent predictor effects or biological causality. No logistic nomogram was constructed for the KNN model.

Baseline characteristics and between-cohort comparisons

For the revised Table 1, age was compared between the 93-patient development cohort and the 40-patient external validation cohort using a two-sided Welch independent-samples t test, without assuming equal variances. Maximum tumor diameter, SII, NLR, PLR, and PNI were compared using two-sided Mann–Whitney U tests with the asymptotic normal approximation, correction for tied ranks, and no continuity correction. Histologic subtype (small-cell versus large-cell neuroendocrine carcinoma) was compared using a two-sided Fisher exact test. Pearson χ^2 tests without continuity correction were used for pathological LNM status, four-category FIGO 2009 stage (I–IV; a 2 × 4 contingency table), biopsy-reported mixed histology, and MRI-assessed parametrial, vaginal, uterine corpus, vesicouterine space, and rectouterine space involvement.

Continuous variables in Table 1 are reported as mean ± sample SD, with sample variances calculated using denominator $n - 1$; categorical variables are reported as number and percentage. For Table 1, pre-existing log10-transformed SII, NLR, and PLR values were back-transformed for each patient as 10^x before original-scale means, SDs, and SMDs were calculated. This descriptive back-transformation left the log10-scale values used for model development and prediction unchanged.

Absolute SMDs were calculated as follows:

Continuous variable: $\text{SMD} = |m_A - m_B| / \sqrt{(s_A^2 + s_B^2)/2}$.

Binary variable: $\text{SMD} = |p_A - p_B| / \sqrt{\{p_A(1 - p_A) + p_B(1 - p_B)\}/2}$.

A and B denote the development and external validation cohorts; m is the cohort mean, s the sample SD, and p the category proportion. Each of the four FIGO stages was compared with all other stages, and the maximum absolute category-specific SMD was reported. These descriptive sample SDs differ from the population-SD scaling parameters used for model standardization. Summaries, tests, and SMDs were calculated from recorded patient-level analysis values or exact category counts before rounding.

Rounded table means, SDs, and percentages were not used as inputs. Recalculation for this revision used Python version 3.11.3 and SciPy version 1.10.1.

Exploratory FFPE single-nucleus RNA sequencing analysis

FFPE single-nucleus RNA sequencing (snRNA-seq) was performed in a matched subset of 8 patients from the radiomics cohort with pretreatment MRI and adequate FFPE tumor tissue. FFPE-compatible transcriptomic libraries were prepared using the 10x Genomics Chromium Fixed RNA Profiling workflow and the manufacturer's protocol for isolating cells from FFPE tissue sections for Chromium Fixed RNA Profiling. Probe hybridization, library construction, and paired-end sequencing followed the recommended Fixed RNA Profiling workflow.

Raw sequencing data were processed using Cell Ranger version 7.1.0. The cellranger multi workflow for Fixed RNA Profiling data handled probe-associated read processing, alignment, barcode assignment, and UMI counting. It generated feature-barcode count matrices and standard quality-control metrics.

Downstream snRNA-seq analysis was performed mainly in R using Seurat version 4.0.0, with quality control performed separately for each sample. Nuclei/barcodes were removed if they met any prespecified low-quality criterion: fewer than 200 detected genes, fewer than 1,000 UMIs, log10GenesPerUMI <0.7, mitochondrial UMI fraction >5%, or hemoglobin gene fraction >5%. log10GenesPerUMI was calculated as log10(detected genes) / log10(total UMIs). Putative doublets were identified and removed within each sample using the DoubletFinder R package version 2.0.3. Expected doublet rates were determined from sample loading and recovered-nuclei estimates.

Count matrices were normalized using Seurat NormalizeData with the LogNormalize method and a scale factor of 10,000. Highly variable genes were identified using FindVariableFeatures with the vst method and 5,000 features. Data were scaled using ScaleData, and principal component analysis was performed using RunPCA. The first 30 principal components were retained for downstream analyses. No covariate regression was performed.

Batch integration used the Harmony R package version 1.2.3, with patient/sample identity as the batch factor. Harmony-corrected embeddings were used to construct the neighbor graph, cluster cells, and

produce two-dimensional visualizations. The nearest-neighbor graph used 15 neighbors, and clustering used the Louvain algorithm in Seurat at a resolution of 0.6. Two-dimensional visualization used t-distributed stochastic neighbor embedding (t-SNE).

For t-distributed stochastic neighbor embedding (t-SNE) visualization, random downsampling yielded 33,223 nuclei, approximately 30% of the total.

Cell types and subtypes were annotated using canonical marker genes, cluster-specific differentially expressed genes, SingleR version 1.4.1 as an auxiliary reference, and manual curation. Major annotation markers included EPCAM and KRT family genes for epithelial/epithelial-like cells; CD3D, CD3E, TRAC, and NKG7 for T/NK cells; LYZ, CD68, CD163, and LST1 for myeloid cells; MS4A1, CD79A, CD79B, MZB1, and JCHAIN for B cells/plasma cells; COL1A1, COL1A2, DCN, and LUM for fibroblasts; and PECAM1 and VWF for endothelial cells.

Because epithelial-like cells are not necessarily malignant, CopyKAT version 1.0.4 was used to infer copy-number variation profiles at the patient level, with immune cells and other non-epithelial cells as references where applicable. These profiles were interpreted alongside marker expression and were not the sole criterion for malignancy. Malignant epithelial cells were identified by integrating CopyKAT-inferred profiles, neuroendocrine-related marker programs, and proliferation-associated expression features. Malignant epithelial subgroups were then annotated by neuroendocrine-related programs, including ASCL1-, NEUROD1-, and YAP1-related programs where applicable.

The patient was the analytical unit for imaging–transcriptomic associations. Analyses covered five major cell classes (epithelial cells, macrophages, fibroblasts, B cells, and T cells) and 38 annotated subtypes. The exported major-class proportions were percentages of all nuclei from the same patient, including the seven major classes in the cellular atlas. The exported subtype proportions were percentages of the combined nuclei from the five analyzed classes, excluding endothelial cells and neutrophils from the denominator. These subtype percentages differ from the within-parent-class percentages used for compositional visualization in Supplementary Figure S5B and Supplementary Table S12. The patient-specific denominators and source proportions are provided in Transcriptomic_Source_Data.zip.

Cell proportions and pathway scores were correlated separately with the intratumoral T1WI-CE and 3-mm peritumoral T1WI-CE+DWI Rad-scores; fusion-model probabilities were not used.

Gene set variation analysis

Gene set variation analysis used KEGG pathway and GO biological-process gene sets in R. Gene sets were loaded with GSEABase version 1.44.0, and GSVA version 1.30.0 was run with kcdf = "Gaussian" for log-normalized expression data. Within each patient and annotated cell population, pathway scores were averaged across nuclear barcodes shared by the annotation table and GSVA matrix. A lack of barcode overlap yielded missing pathway scores; missing per-nucleus scores were excluded from the corresponding mean. Patient-level aggregation imposed no additional minimum-nucleus cutoff. Matching counts are reported for each patient, population, and database. The barcode-matching summaries recorded complete matches for all annotated nuclei; missing patient-population means occurred when no nuclei were annotated to that population. For the displayed antigen-presenting macrophage pathways, P5 had no annotated or matched nuclei, yielding seven evaluable patients. This absence among the analyzed nuclei was not interpreted as proof of biological absence. The contributing nuclear counts varied across patients; for example, the antigen-presenting macrophage pathway means for P3 were based on one nucleus.

Patient-level association analysis and post hoc display selection

For each regional score and measurement, complete finite patient pairs were identified without imputation or replacement of missing pathway values by zero. Correlations with fewer than seven complete pairs were recorded as not tested. For eligible comparisons, Spearman ρ was calculated from midranks. Two-sided P values were computed from all n! permutations of patient labels for one variable, retaining the actual tied-rank structure and counting permutations with $|\rho\_permuted| \geq |\rho\_observed|$. Thus, 5,040 permutations were used for n = 7 and 40,320 for n = 8. The reported P values are exact for this permutation procedure and are not the previous asymptotic P values or probabilities inferred from rounded coefficients under a no-ties assumption.

The exported data comprised 370,646 measurements per regional score: 370,603 cell-population–pathway combinations and 43 cell proportions. After requiring at least seven complete patient pairs, 232,726 correlations were eligible per regional score (43,078 at the major-class level and 189,648 at the subtype level). The final reporting applied Benjamini–Hochberg correction separately within each regional score across all 232,726 eligible comparisons, combining both annotation levels, all populations, GO and KEGG pathways, and cell proportions before significance filtering. The original run outputs had applied BH separately to major-class and subtype analyses; these original values are retained for traceability alongside the combined-family q values in the source data. Neither approach identified an association with $q < 0.05$. There were 5,584 intratumoral and 7,567 peritumoral correlations with unadjusted exact $P < 0.05$; these nominal thresholds do not define multiplicity-adjusted discoveries.

The 11 cell–pathway pairs were originally selected post hoc for concordance between regional scores and functional coherence, following extensive screening of immune, stromal, hypoxia/vascular, and metabolic themes. They were retained unchanged in this revision for transparency rather than selected anew on the recalculated P values. Supplementary Table S13 reports their recomputed coefficients, effective sample sizes, exact P values, and combined-family BH q values. The patient-level source data contain both regional Rad-scores and the corresponding pathway means, including missing entries and contributing nuclear counts. Complete results for all screened measurements, including nonsignificant and ineligible entries, are also supplied. No pathway-enrichment test or LNM mechanism is inferred from this selected set.

The displayed subset comprised 11 cell–pathway pairs: endocytosis, proteasome, antigen processing and presentation, and TAP-dependent MHC-I presentation of exogenous peptides in antigen-presenting macrophages; antigen processing and presentation and phagosome–lysosome fusion in UPR-like macrophages; chemokine and TNF signaling in T cells; cadherin-mediated cell–cell adhesion in fibroblasts; calcium-dependent cell–matrix adhesion in inflammatory fibroblasts; and cell–cell adhesion in matrix-producing ECM-high fibroblasts. Full pathway names and KEGG/GO identifiers for Supplementary Figure S5C,D are provided in Supplementary_Figure_S2_AB_plot_data.csv within Transcriptomic_Source_Data.zip; the filename retains the earlier figure numbering.

Supplementary Figure S5C,D displays the same 11 cell-pathway pairs and their six corresponding cell proportions for both regional scores. Color encodes Spearman ρ, with red indicating positive correlations and blue indicating negative correlations. Circle area is proportional to $-3 \times \log10$ of the unadjusted exact permutation P value; neither color nor size denotes FDR significance. No comparisons by pathological LNM status were used to select the displayed examples.

Missing data and software

All candidate predictors were complete in the final radiomics modeling cohort, so no imputation was performed. Patients were excluded under the prespecified eligibility criteria for missing pathological nodal status, missing essential pretreatment MRI sequences, non-diagnostic MRI, or incomplete key clinical/pathological information. These exclusions are reported in the study flow diagram.

Revised model development used Python version 3.6.15, NumPy 1.19.5, pandas 1.1.5, scikit-learn 0.24.2, SciPy 1.5.3, and joblib 0.17.0. These versions were recorded in the modeling environment file. Segmentation and registration used 3D Slicer version 5.8.1, and radiomics extraction used PyRadiomics version 3.1.0. Descriptive statistical analyses used R version 4.3.1. Additional comparisons calculated from locked predictions were verified using Python version 3.12.14. Calibration diagnostics and decision curves were initially calculated using the revised Python analysis scripts. The final calibration, decision-curve, and KNN-explanation figures were generated and independently checked in R version 4.6.1.

Software for snRNA-seq analysis included Cell Ranger version 7.1.0 for raw FFPE snRNA-seq data processing; Seurat version 4.0.0 for downstream snRNA-seq analysis; Harmony R package version 1.2.3 for batch integration; DoubletFinder version 2.0.3 for doublet detection; SingleR version 1.4.1 for auxiliary cell-type annotation; CopyKAT version 1.0.4 for copy-number inference; GSEABase version 1.44.0 for gene-set loading; and GSVA version 1.30.0 for pathway activity analysis. Databases, reference files, and software versions used for snRNA-seq analysis are summarized in Supplementary Table S6. The revised patient-level correlation runs used Python 3.10.19, NumPy 2.2.6, pandas 2.3.3, SciPy 1.15.2, and openpyxl 3.1.5, as recorded in their run manifests. Spearman coefficients were computed with scipy.stats.spearmanr, and exact permutation P values were calculated separately by full enumeration as

described above. An independent calculation from the exported paired data reproduced all eligible coefficients, effective sample sizes, exact permutation P values, and original BH q values. Combined-family BH values were calculated from the complete set of eligible exact P values, and the verification script is included with the source data.
